# The effectiveness of mind mapping versus lecture-based learning in medical education of China’s standardized residency training: a systematic review and meta-analysis of randomized controlled studies

**DOI:** 10.3389/fmed.2026.1789650

**Published:** 2026-05-05

**Authors:** Dongjun Dai, Yuzhi Fan, Qiang Yan, Qichun Wei

**Affiliations:** 1 Department of Radiation Oncology, The Second Affiliated Hospital, Zhejiang University School of Medicine, Hangzhou, Zhejiang, China; 2 Cancer Center, The Affiliated Huzhou Hospital, Zhejiang University School of Medicine (Huzhou Central Hospital), Huzhou, China

**Keywords:** China’s standardized residency training, clinical competence, examination scores, lecture-based learning, meta-analysis, mind mapping, systematic review

## Abstract

**Background:**

Mind mapping has been widely used as an intervention in medical education of China’s standardized residents training (SRT) program. Our study aims to investigate the effectiveness of mind mapping compared with lecture-based learning (LBL) in China’s SRT.

**Methods:**

A PRISMA guideline based systemic review and meta-analysis (PROSPERO registration number: CRD420251244861) was performed. We searched the publications in PubMed, China National Knowledge Infrastructure, VIP database and Wanfang database up to December 24, 2025 to identify randomized controlled studies with outcomes measure from mind mapping and LBL. Two independent reviewers performed the study selection, data extraction and quality assessment. All the statistical analyses were performed by R 4.1.1 software.

**Results:**

A total of 52 studies with 3,312 participants (Mind mapping group = 1,698, LBL group = 1,614) were selected in our meta-analysis. These studies published from 2017 to 2025. Meta-analysis identified significantly higher examination scores in mind mapping group compared to LBL group in theoretical knowledge (SMD = 1.45, 95% CI: 1.16–1.74), case analysis (SMD = 1.34, 95% CI: 0.95–1.72), and procedural skill (SMD = 1.68, 95% CI: 1.23–2.13). Questionnaire surveys results showed that residents were more satisfied with the mind mapping and had improvements in level of theoretical knowledge, learning motivation, problem-solving ability, proficiency in literature retrieval, clinical skills, and teamwork. There were 98% of the included studies lacked allocation concealment and blinding, and high heterogeneity (I^2^ > 80%) was present in 11 out of 12 meta-analyses using continuous data. GRADE assessment showed that the overall certainty of evidence was very low.

**Conclusion:**

Our meta-analysis indicates that mind mapping is potentially more effective than LBL in medical education of China’s SRT system. Given the limitations of substantial heterogeneity and very low certainty evidence, our findings are insufficient for strong practice recommendations. Further well-designed studies are required to confirm our findings.

**Systematic review registration:**

https://www.crd.york.ac.uk/PROSPERO/view/CRD420251244861.

## 1 Introduction

Postgraduate education facilitates the bridge between historical practices and future specialty developments (1). The complexity and heterogeneity of medical diseases and clinical scenarios demand for high-quality postgraduate education (2). In China, the nationwide Standardized Residency Training (SRT) program was launched in 2013, aiming to enhance resident physicians’ professional competence and improve overall quality (3). However, many training bases in China still utilize traditional lecture-based learning (LBL) method, which is often associated with passive learning among residents and may impair their ability to integrate theoretical knowledge with clinical practice. Although various teaching methods have been introduced into China’s SRT system (3), there remains a demand for more effective pedagogical models.

Mind mapping is a cognitive tool that organizes information visually using a graphical structure. It typically begins with a central image representing the main topic and then extends major branches radiating from the center, with each labeled with a keyword representing a core subtopic (4). The mind mapping allows the learner to create the best arrangement of information, enhancing the comprehension, memory efficiency and cooperative ability, which ultimately promotes ideation and critical thinking (5, 6). In addition, as a teaching resource, it could be effectively used with other teaching methods such as problem-based learning, small-group teaching (7).

Mind mapping has been widely used in medical education and has been found to increase both examination scores and clinical competence (6, 8, 9). Recently, several randomized controlled studies comparing the effects of mind mapping and LBL have been published in China. Among them, some employed the mind mapping alone (10–14), while others combined it with other innovative teaching methods (15–19). Many of these studies reported that mind mapping outperformed LBL in various outcomes. However, these studies are often limited by small sample sizes.

Given that no prior meta-analysis or systematic review has focused on mind mapping in China’s SRT, we conducted the present study to compare the effectiveness of mind mapping versus LBL in terms of examination performance and clinical competencies, by synthesizing available evidence from relevant randomized controlled studies.

## 2 Methods

### 2.1 Data collection

Our study was conducted in accordance with the Preferred Reporting Items for Systematic reviews and Meta-Analyses (PRISMA) 2020 guidelines (20) (Supplementary Data 1). The protocol of our study was registered in the International Prospective Register of Systematic Reviews (PROSPERO) (CRD420251244861). The literature search was performed up to December 24, 2025 across PubMed and three major Chinese databases that included China National Knowledge Infrastructure, VIP database and Wanfang database. There was no language restriction in literature screening.

### 2.2 The inclusion and exclusion criteria

Study eligibility was defined using the PICOS (Population, intervention, comparison, outcome, study design) framework (Supplementary Data 2). The inclusion criteria comprised: (1) population: postgraduate medical residents with no restrictions on the specialties; (2) intervention: implementation of mind mapping as a single intervention or combined with other innovative education method; (3) comparison: use of traditional teacher-centered LBL as control group; (4) outcome: the study should provide post-intervention assessment data from examinations or questionnaire surveys; (5) study design: randomized controlled study. The exclusion criteria comprised: (1) studies included undergraduate students or nurse students or non-clinical residents such as pharmacy students; (2) studies that did not include mind mapping as an intervention; (3) studies lacking a concurrent control group, such as single-arm pretest-posttest studies; (4) studies without outcome measurement; (5) studies without a randomized design.

### 2.3 Literature screening and the quality assessment

The literature screening process followed the PRISMA 2020 flow diagram for new systematic reviews which included searches of databases and registers only (20). After reading the titles and abstracts from different database and removing the duplicate records, potential full-text articles were retrieved for further assessment under the eligibility criteria. Studies were then finally selected. The assessment of the quality of each included study was conducted through Cochrane risk-of-bias tool of randomized trials (21), which comprised randomization sequence generation, allocation concealment, blinding of participants and personnel, blinding of outcome assessment, incomplete outcome data, selective reporting and other bias. The assessment of each item was marked as “low risk,” “high risk” or “unclear” according to the description of each study.

The literature screening and study quality assessment were performed by two independent reviewers (DD and YF). Any discrepancies during selection or quality assessment were resolved through discussion that involved a third reviewer (QW).

The certainty of evidence was rated using the Grading of Recommendations Assessment, Development and Evaluation (GRADE) framework (22), with evaluation across five domains: risk of bias, inconsistency, indirectness, imprecision, and publication bias. The GRADE certainty levels of evidence are defined as “high,” “moderate,” “low,” or “very low” according to the GRADE Handbook (22). We regarded evidence from randomized controlled studies as initially high certainty, and downgraded the evidence by one level for serious limitations and two levels for very serious limitations across the five GRADE domains.

### 2.4 Data extraction

Two investigators (DD and YF) independent extracted the following items from the selected studies: first author/year, department, sample size, baseline characteristics matched between groups, faculty matching information between groups, intervention methods, how the mind mapping conducted, mind mapping course frequency, whether the course frequency matched between groups, outcome measures and intervention duration. There were no assumptions made about any missing or unclear information.

### 2.5 Statistical analysis

All the statistical analyses were performed in R 4.1.1 software.^1^ The inter-rater reliability (kappa coefficient) was calculated by “cohen.kappa” function from “psych” package. All the meta-analysis related statistics were conducted by “meta” package. For continuous outcomes, the “metacont” function was used to calculate pooled standardized mean differences (SMD) and 95% confidence intervals (CIs) with Hedges’ g method (23). For binary outcomes, the “metabin” function was used to calculate pooled odds ratio (OR) and 95%CI with restricted maximum likelihood method (24). Meta-analyses were performed only for outcomes reported with at least three studies. Given the expected inherent heterogeneity in intervention protocols and outcome assessment criteria among eligible studies, the random-effects model was applied for data pooling. This model yields conservative summary estimates and broader CIs, thereby preventing overinterpretation of the overall effect. The synthetic results were visualized by forest plots. Statistical heterogeneity was quantified using the I^2^ and tau^2^ tests. The prediction intervals would be calculated if I^2^ > 75%. The “metainf” function was used to perform sensitivity analysis to find potential heterogeneity and bias. We also additionally performed a sensitivity analysis excluding studies with high or unclear risk of bias in randomization. If the sensitivity analysis failed to identify the source of the heterogeneity, subgroup analyses and meta-regression were also used to detect heterogeneity in meta-analyses with sufficient studies (*n* ≥ 10). The “metareg” function was used to perform the meta-regression. The “metabias” function was applied to evaluate the publication bias with Egger’s test (25). If significant publication bias was detected, the trim-and-fill method was applied to adjust the estimate. A two-sided *p*-value < 0.05 was considered statistically significant.

## 3 Results

### 3.1 Literature screening

**Figure 1:**
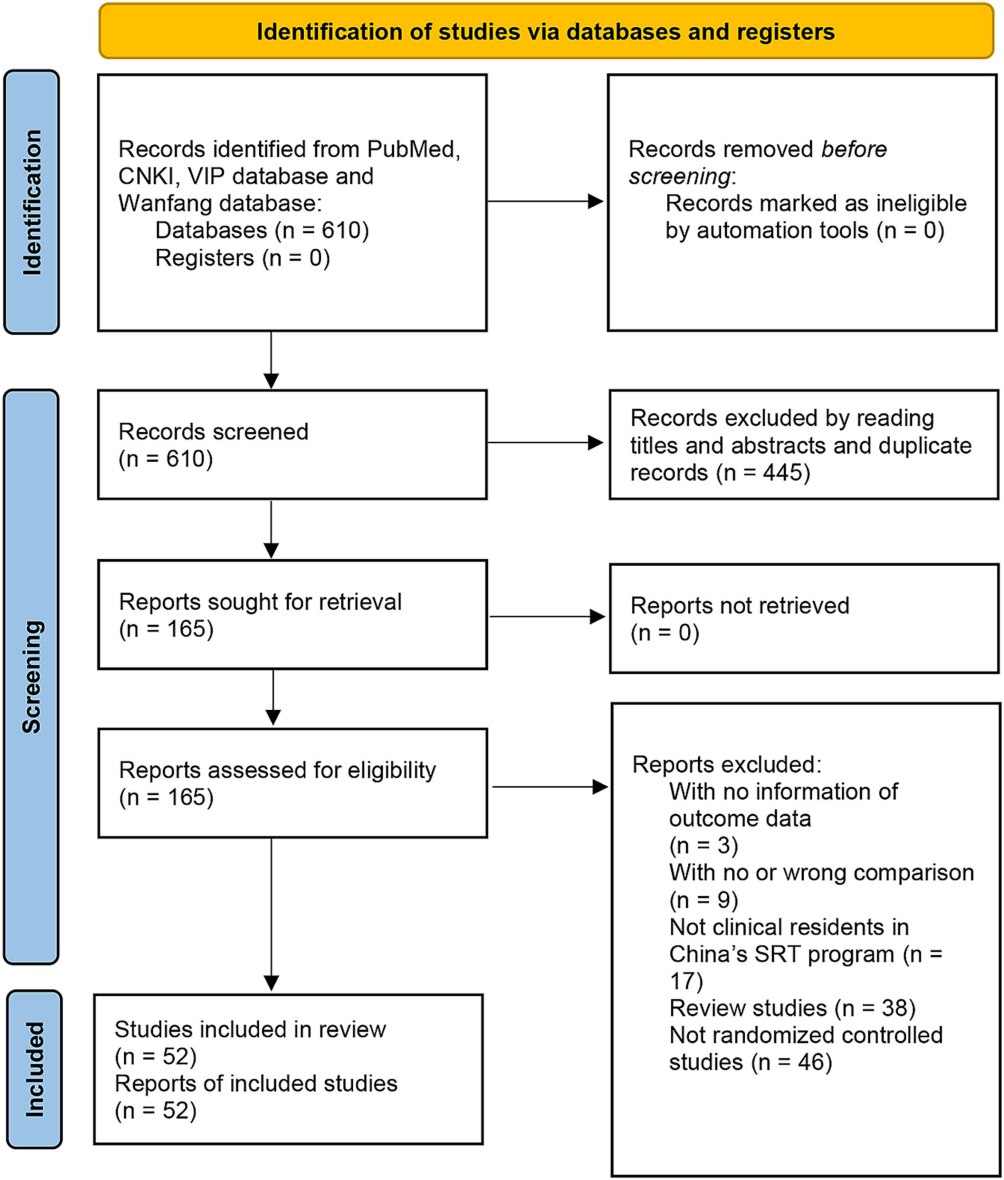
Flowchart of selection process in the meta-analyses.

Figure 1 summarized the study selection process. There were 610 records collected from PubMed, China National Knowledge Infrastructure, VIP database and Wanfang database. After reading the titles and abstract and removing the duplicate records, we retrieved 165 full-text articles. Among them, we excluded 3 studies with no information of outcome data, 9 studies with no or wrong comparison, 17 studies included no clinical residents of China’s SRT, 38 review studies and 46 studies that were not randomized controlled studies. Finally, there were 52 eligible studies included in our study. The calculated Cohen’s kappa coefficients were 0.76 (95%CI: 0.71–0.82) for title and abstract screening and 0.74 (95%CI: 0.66–0.82) for full-text screening.

### 3.2 Characteristics of included studies

The key characteristics of the 52 (8–19, 26–65) included studies were presented in Table 1. There were 49 studies in Chinese and 3 studies in English. These studies published between 2017 and 2025 with totally 3,312 participants (Mind mapping group = 1,698, LBL group = 1,614). The median sample size of the included studies was 57.5, with an interquartile range of 40 to 72.75. The residents’ groups were matched at baseline for variables such as age, gender, educational background, and entrance examination scores. The studies originated from departments of internal medicine (*n* = 25), surgery (*n* = 11), diagnostic imaging (*n* = 9), anesthesiology (*n* = 5), and departments that not specified (*n* = 2). There were 22 studies used single mind mapping as intervention while the other 30 studies combined mind mapping with other education methods such as PBL, CBL, simulation and web or app-based method. The mind map during the course was generated by students (*n* = 32), faculty member (*n* = 5) or both (*n* = 15). Nineteen studies mentioned the faculty matching information between groups, there were 18 studies used the same faculty members in two groups while one study showed that it matched the teaching experience between two groups. Eleven studies reported mind mapping course frequency, with 9 studies conducting courses weekly and 2 studies conducting courses monthly. Eleven studies reported that they matched the frequency between two groups. Eighteen studies reported intervention duration that ranged from 2 weeks to 6 months.

**Table 1:** The characteristics of the included studies in current meta-analysis.

First author/Year	Department	Sample size (I/C)	Interventions	Residents matched for	Faculty matching	Mind mapping course structure	Intervention frequency	Control group matching	Duration of intervention	Outcome
Yuan Cheng/2019 (65)	Anesthesiology	154 (76/78)	Mind mapping	EB	NA	Co-constructed	NA	NA	NA	Exam
Yunfeng Jiang/2024 (26)	Surgery	60 (30/30)	Mind mapping and video	Age, gender	NA	Faculty-generated	NA	NA	NA	Exam, QS
Shanshan Cui/2024 (27)	Anesthesiology	46 (23/23)	Mind mapping and SP	Age, gender, BES	Same faculty member	Student-generated	NA	NA	NA	Exam, QS
Zhenyu Shen/2023 (10)	Surgery	34 (17/17)	Mind mapping	Age, gender, BES	NA	Faculty-generated	Monthly	Matched	6 months	Exam, QS
Yingbiao Zhu/2023 (11)	Internal Medicine	186 (93/93)	Mind mapping	Age, gender, EB	NA	Student-generated	NA	NA	1 month	Exam
Jiajia Fan/2021 (28)	Internal Medicine	40 (20/20)	Mind mapping and CBE	Age, gender	NA	Student-generated	Weekly	NA	3 months	Exam, QS
Yi Guo/2020 (29)	Diagnostic imaging	22 (11/11)	Mind mapping and FC	Age	NA	Co-constructed	NA	NA	NA	Exam, QS
Min Lv/2025 (30)	Surgery	40 (20/20)	Mind mapping and virtual simulation	Age, gender, EB, BES	NA	Student-generated	NA	NA	NA	Exam, QS
Jun Liu/2025 (31)	Internal Medicine	80 (40/40)	Mind mapping and CDIO	Age, gender, EB	Same faculty member	Co-constructed	NA	NA	NA	Exam, QS
Dandan Ma/2024 (12)	Surgery	169 (85/84)	Mind mapping	Age, gender	Same faculty member	Co-constructed	NA	NA	1 month	Exam, QS
Li Qu/2023 (32)	Internal Medicine	46 (23/23)	Mind mapping and scene simulation	Age, gender,	NA	Student-generated	NA	NA	NA	Exam
Hongjuan Shi/2023 (13)	Internal Medicine	60 (30/30)	Mind mapping	Age, gender, BES	NA	Co-constructed	NA	NA	NA	Exam, QS
Liudan Tu/2023 (14)	Internal Medicine	75 (39/36)	Mind mapping	Age, gender, EB	Same faculty member	Co-constructed	Monthly	Matched	3 months	Exam
Shuo Wu/2022 (33)	Surgery	46 (23/23)	Mind mapping	Age, gender, BES	Same faculty member	Student-generated	Weekly	Matched	3 months	Exam, QS
Xin Liao/2022 (34)	Diagnostic imaging	80 (40/40)	Mind mapping	Age, gender	NA	Student-generated	NA	NA	NA	Exam, QS
Le Zhang/2021 (15)	Internal Medicine	28 (14/14)	Mind mapping and PBL	Age, gender	NA	Student-generated	NA	NA	1–2 months	Exam, QS
Yan Xu/2018 (35)	Diagnostic imaging	30 (15/15)	Mind mapping and online lecture	Age, gender	NA	Co-constructed	NA	NA	NA	Exam, QS
Jing Zhao/2017 (36)	Not specified	59 (30/29)	Mind mapping and microteaching	Age, gender, BES	Same faculty member	Faculty-generated	NA	Matched	NA	Exam, QS
Jinyu Zhang/2025 (16)	Internal Medicine	59 (35/24)	Mind mapping and PBL	Age, gender	Same faculty member	Student-generated	Weekly	Matched	1 month	Exam, QS
Ying Zhou/2025 (37)	Internal Medicine	40 (20/20)	Mind mapping and mobile platform	NA	Same faculty member	Student-generated	Weekly	Matched	2 months	Exam, QS
Qian Liu/2025 (38)	Internal Medicine	46 (23/23)	Mind mapping	Age, gender	NA	Student-generated	NA	NA	NA	Exam, QS
Lin Guo/2025 (39)	Diagnostic imaging	64 (32/32)	Mind mapping	Age, gender	NA	Co-constructed	NA	NA	NA	Exam, QS
Xiaoxue Zhang/2025 (40)	Internal Medicine	34 (17/17)	Mind mapping	Age	NA	Co-constructed	NA	NA	NA	Exam, QS
Yue Yu/2024 (41)	Internal Medicine	50 (25/25)	Mind mapping and CPBL	Age, gender	NA	Student-generated	NA	NA	NA	Exam, QS
Ning Du/2024 (42)	Anesthesiology	60 (30/30)	Mind mapping	Age, gender, EB	Same faculty member	Co-constructed	NA	NA	NA	Exam, QS
Yanming Zhang/2024 (43)	Internal Medicine	80 (40/40)	Mind mapping	Age, gender	NA	Student-generated	Weekly	Matched	NA	Exam, QS
Jia Chen/2024 (44)	Internal Medicine	26 (13/13)	Mind mapping and 3D anatomy software	Age, gender	NA	Student-generated	NA	NA	3 months	Exam, QS
Yipeng Liu/2024 (45)	Anesthesiology	60 (30/30)	Mind mapping	Age, gender, EB	Same faculty member	Co-constructed	NA	NA	NA	Exam, QS
Min Tang/2024 (46)	Internal Medicine	56 (28/28)	Mind mapping and R2C2	Age, gender	NA	Co-constructed	NA	NA	NA	Exam, QS
Jin Zhang/2024 (17)	Diagnostic imaging	66 (33/33)	Mind mapping and CBL	Age, gender, BES	Same faculty member	Student-generated	NA	NA	2 months	Exam, QS
Yanqian Deng/2024 (47)	Surgery	100 (50/50)	Mind mapping and interview style teaching	Age, gender	NA	Faculty-generated	NA	NA	3 months	Exam, QS
Wenjie Xu/2024 (18)	Internal Medicine	60 (30/30)	Mind mapping and PBL	Age, gender	NA	Faculty-generated	NA	NA	NA	Exam, QS
Jingmin Dong/2023 (48)	Diagnostic imaging	40 (20/20)	Mind mapping and CBL	Age, gender	Same faculty member	Student-generated	Weekly	Matched	2 months	Exam, QS
Lingtao Liu/2023 (49)	Surgery	30 (15/15)	Mind mapping	Age, gender, EB, BES	Same faculty member	Student-generated	Weekly	Matched	3 months	Exam, QS
Fan Liu/2023 (19)	Internal Medicine	32 (16/16)	Mind mapping and CBL	Age, gender	NA	Student-generated	NA	NA	NA	Exam, QS
Shengqun Jiang/2022 (50)	Surgery	48 (24/24)	Mind mapping and PBL	Age, gender	NA	Student-generated	NA	NA	NA	Exam, QS
Yafang Wei/2022 (51)	Internal Medicine	80 (40/40)	Mind mapping and FC	Age, gender, BES	Same faculty member	Co-constructed	NA	NA	NA	Exam
Hongyan Li/2022 (52)	Surgery	41 (19/22)	Mind mapping and CBL	Age, gender	Same faculty member	Student-generated	NA	NA	NA	Exam, QS
Bing Zhou/2021 (53)	Surgery	98 (49/49)	Mind mapping	Age, gender, EB, BES	TE	Co-constructed	NA	NA	NA	Exam, QS
Shuai Fu/2021 (54)	Diagnostic imaging	30 (15/15)	Mind mapping	Age, gender	NA	Student-generated	NA	NA	NA	Exam, QS
Li Yao/2019 (55)	Internal Medicine	125 (63/62)	Mind mapping and PBL	Age, gender, EB, BES	NA	Student-generated	NA	NA	NA	Exam, QS
Zhouwei Xu/2025 (9)	Internal Medicine	216 (144/72)	Mind mapping and CPBL	Age, gender, EB, BES	NA	Student-generated	NA	NA	At least 2 weeks	Exam, QS
Rong Liu/2025 (8)	Diagnostic imaging	42 (21/21)	Mind mapping and CBL + Mini-CEX	Age, gender	NA	Student-generated	NA	NA	1 month	Exam, QS
Yao Hu/2025 (56)	Internal Medicine	60 (30/30)	Mind mapping	Age, gender, EB, BES	Same faculty member	Student-generated	NA	NA	2 months	Exam, QS
Jianping Gong/2023 (57)	Anesthesiology	60 (30/30)	Mind mapping and PBL	Age, gender	NA	Student-generated	NA	NA	NA	Exam, QS
Wenjuan Wang/2020 (58)	Internal Medicine	72 (36/36)	Mind mapping and mobile platform	Age, gender	NA	Student-generated	Weekly	NA	NA	Exam, QS
Shushu Zhang/2024 (59)	Not specified	100 (50/50)	Mind mapping	Age, gender, EB	Same faculty member	Student-generated	NA	Matched	NA	Exam, QS
Dongzhu Zhang/2019 (60)	Diagnostic imaging	10 (5/5)	Mind mapping	Age, gender	NA	Co-constructed	NA	NA	NA	Exam, QS
Yuehua Pu/2017 (61)	Internal Medicine	26 (13/13)	Mind mapping	Age, gender, EB	NA	Student-generated	NA	NA	2 months	Exam, QS
Jun Li/2023 (62)	Internal Medicine	60 (30/30)	Mind mapping and online education	Age, gender,	Same faculty member	Student-generated	NA	NA	NA	Exam, QS
Qingsong Zhang/2025 (63)	Surgery	40 (20/20)	Mind mapping	Age, gender,	NA	Student-generated	Weekly	Matched	NA	Exam, QS
Yuanhui Sun/2025 (64)	Internal Medicine	46 (23/23)	Mind mapping and CPBL	Age, gender, BES	NA	Student-generated	NA	NA	NA	Exam, QS

I, intervention group; C, control group; SP, standardized patients; CBE, competency-based education; FC, flapped classroom; CDIO, Conceive–Design–Implement–Operate approach; PBL, problem-based learning; CPBL, case and problem-based learning; 3D print, three-dimensional print; R2C2, Relationship, Reaction, Content, Coaching; CBL, case-based learning; BES, basic examination scores; EB, educational background; NA, not available; TE, teaching experience; QS, questionnaire surveys.

Studies utilized post-learning examinations as an outcome measure that included theoretical knowledge (*n* = 41), case analysis (*n* = 18), and procedural skills (*n* = 28). Additionally, there were 47 studies applied questionnaire surveys and reported continuous or binary outcomes including level of theoretical knowledge, clinical reasoning, learning motivation, autonomous learning ability, problem-solving ability, proficiency in literature retrieval, clinical skills, teamwork, and course satisfaction.

### 3.3 Assessment of the study quality

**Figure 2:**
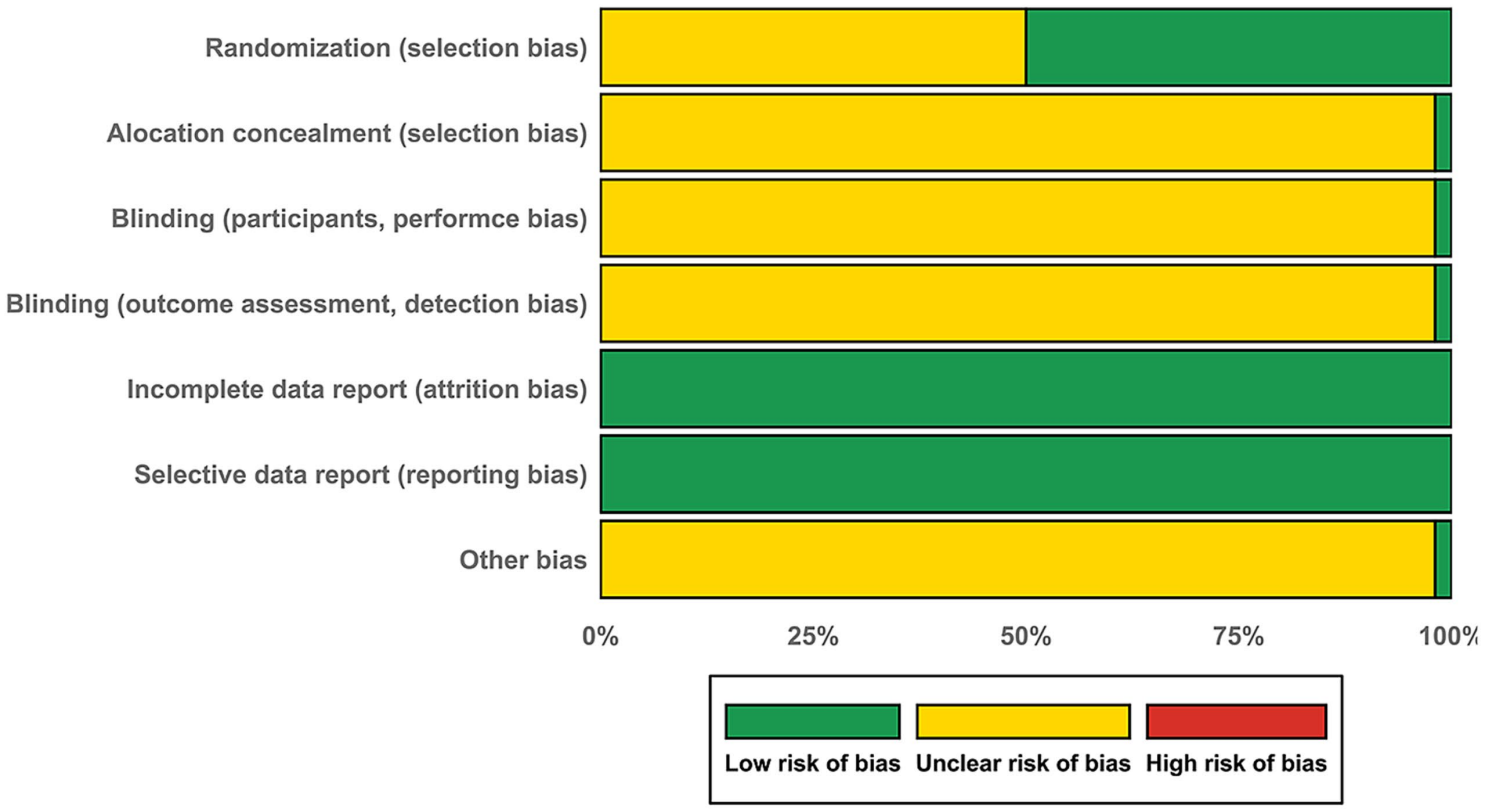
Risk of bias graph of the current meta-analysis. The vertical axis represents the risk of bias items, and the horizontal axis represents the percentages of all included studies.

The risk assessment of our meta-analysis was shown in Figure 2. The detailed results of each study’s quality assessment were shown in Supplementary Table S1. Among the 52 included studies, there were 26 studies described their randomization process. Only 1 of the 52 studies (2%) reported adequate allocation concealment, and only 1 of the 52 (2%) adopted blinding measures, indicating a high risk of selection bias and performance bias. All included studies reported complete outcome data, with all outcomes mentioned in the methods sections adequately reported in the results sections. Based on this consistency between pre-specified and reported outcomes, we assessed the risk of selective outcome reporting as low for all studies. For the other bias, only one study (9) provided adequate information of randomization, allocation concealment and blinding, together with fully reported baseline comparability which included age, gender, basic examination score and educational background. This study was judged to have low risk of other bias. For all remaining studies, consider their study design limitations, we rated them as having unclear risk of other bias.

### 3.4 Effects of mind mapping on examination scores

**Figure 3:**
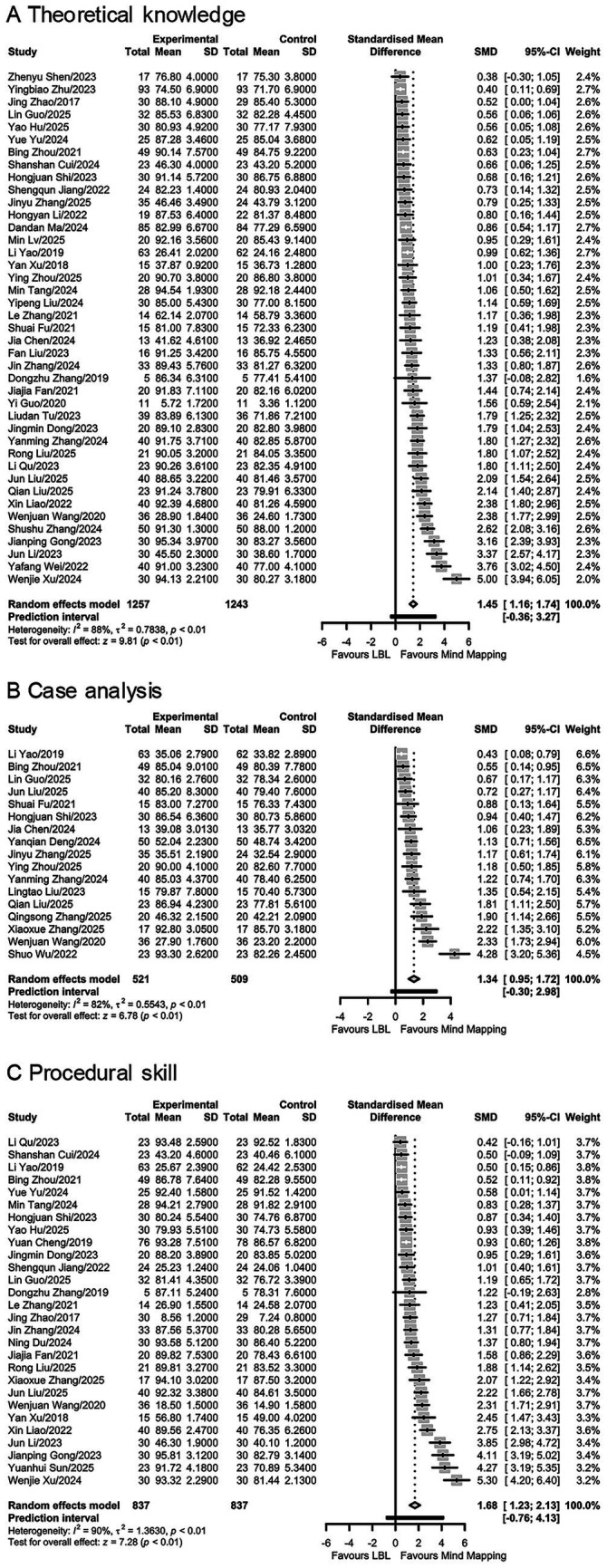
Forest plots depicting meta-analyses on the effect of mind mapping on examination scores. The meta-analyses on the effect of mind mapping on the **(A)** theoretical knowledge scores, **(B)** case analysis scores, and **(C)** procedural skill scores. The large diamond at the bottle of the plot represents the pooled SMD of all studies. The width of the diamond represents with 95% CI. The black horizontal bar represents the 95% prediction interval.

As shown in Table 2 and Figure 3, compared with LBL, Meta-analysis showed that mind mapping significantly improved examination scores on theoretical knowledge (41 studies, SMD = 1.45, 95% CI, 1.16–1.74, *p* < 0.001; I^2^ = 87.7%, tau^2^ = 0.784), case analysis (18 studies, SMD = 1.34, 95% CI, 0.95–1.72, p < 0.001; I^2^ = 82.5%, tau^2^ = 0.554), and procedural skill (27 studies, SMD = 1.68, 95% CI, 1.23–2.13, p < 0.001; I^2^ = 90.1%, tau^2^ = 1.363) (Table 2; Figure 3). Notably, all prediction intervals crossed zero in these meta-analyses (Figure 3). The sensitivity analysis was performed after detecting significant heterogeneity (I^2^ > 50%), however, it did not find the heterogeneity source (Supplementary Figure S1; Supplementary Table S2). As shown in Supplementary Figure S2, further subgroup analyses by departments, interventions mind mapping method, and intervention duration revealed that the heterogeneity decreased to a non-significant level in theoretical knowledge within the surgery department (I^2^ = 0%), case analysis within diagnostic imaging department (I^2^ = 0%) and group receiving intervention of less than 3 months (I^2^ = 0%), procedural skill within the surgery department (I^2^ = 43%) and group receiving intervention of less than 3 months (I^2^ = 20%). Significant heterogeneity persisted in other subgroups (I^2^ > 50%). The mind mapping remained superior to LBL in most subgroup analyses, except for theoretical knowledge within the unspecified departments (SMD = 1.57, 95% CI: −0.48–3.62) and in faculty-generated group (SMD = 1.94, 95% CI: −1.01–4.88), procedural skill in the faculty-generated group (SMD = 3.26, 95% CI: −0.69–7.21). Meta-regression analyses revealed no statistically significant moderators for the observed heterogeneity in meta-analyses on examination scores (*p* > 0.05; Supplementary Table S3). As shown in Table 2, Egger’s tests identified significant publication bias for all examination scores outcomes (*p* < 0.05). Application of the trim-and-fill method eliminated the publication bias (*p* > 0.05) while the meta-analyses after the adjustment still supported the benefit of mind mapping for theoretical knowledge (SMD = 0.84, 95% CI: 0.48–1.21), case analysis (SMD = 0.87, 95% CI: 0.37–1.36) and procedural skill (SMD = 0.95, 95% CI: 0.37–1.53). As shown in Supplementary Table S4, the certainty of the meta-analyses was rated as very low according to the GRADE assessment.

**Table 2:** The meta-analyses results of mind mapping on examination scores and questionnaire surveys compared to LBL.

Items	Study numbers	I	C	SMD/OR^a^ (95%CI)	*p* value	I^2^	tau^2^	Egger’s test	SMD/OR^a^ (95%CI) after TF	*p* value after TF	I^2^ after TF	tau^2^ after TF	Egger’s test after TF
Continuous outcomes													
Theoretical knowledge scores	41	1,257	1,243	1.45 (1.16–1.74)	<0.001	87.7%	0.784	<0.001	0.84 (0.48–1.21)	<0.001	93.0%	1.847	0.597
Case analysis scores	17	521	509	1.34 (0.95–1.72)	<0.001	82.5%	0.554	0.001	0.87 (0.37–1.36)	0.001	88.6%	1.362	0.709
Procedural skill scores	28	837	837	1.68 (1.23–2.13)	<0.001	90.1%	1.363	<0.001	0.95 (0.37–1.53)	0.001	94.0%	3.104	0.684
Level of theoretical knowledge	9	374	301	1.09 (0.63–1.55)	<0.001	80.3%	0.41	0.180					
Clinical reasoning	13	483	400	1.96 (0.91–3.00)	<0.001	90.0%	3.529	0.001	1.01 (−0.34–2.36)	0.143	93.5%	7.844	0.802
Learning motivation	15	519	446	1.80 (1.02–2.58)	<0.001	87.1%	2.233	0.067					
Autonomous learning ability	9	249	248	1.82 (0.56–3.09)	0.005	89.6%	3.555	0.004	0.94 (−0.60–2.48)	0.232	92.7%	7.167	0.885
Problem solving ability	11	270	270	2.32 (1.19–3.46)	<0.001	88.8%	3.486	0.001	1.79 (0.19–3.39)	0.028	92.3%	7.748	0.491
Proficiency in literature retrieval	4	97	97	1.24 (0.63–1.86)	<0.001	65.1%	0.265	0.132					
Clinical skills	7	163	163	1.48 (0.66–2.29)	<0.001	90.2%	1.078	0.023	0.96 (0.00–1.92)	0.049	92.5%	2.012	0.516
Teamwork	7	293	871	2.15 (0.27–4.03)	0.025	94.4%	6.259	0.828					
Course satisfaction	12	392	320	1.93 (0.87–3.00)	<0.001	90.1%	3.332	0.050					
Binary outcomes													
Level of theoretical knowledge	8	234	236	3.91 (2.26–6.78)	<0.001	0.0%	0	<0.001	3.29 (1.90–5.67)	<0.001	9.9%	0	0.680
Clinical reasoning	9	261	252	4.46 (2.85–6.97)	<0.001	0.0%	0	0.001	3.66 (2.37–5.67)	<0.001	0.0%	0	0.777
Learning motivation	10	337	325	3.95 (2.43–6.42)	<0.001	14.1%	0.059	0.006	3.31 (2.10–5.23)	<0.001	23.0%	0.027	0.808
Autonomous learning ability	6	178	170	5.40 (3.16–9.24)	<0.001	0.0%	0	0.020	4.84 (2.77–8.45)	<0.001	0.0%	0	0.976
Problem solving ability	3	110	110	3.55 (0.48–26.27)	0.007	36.1%	0.271	0.416					
Proficiency in literature retrieval	4	146	145	4.87 (2.35–10.08)	<0.001	0.0%	0	0.330					
Clinical skills	4	156	145	4.23 (2.67–6.71)	<0.001	0.0%	0	0.112					
Course satisfaction	14	399	399	5.38 (4.30–6.74)	<0.001	0.0%	0	0.001	4.66 (3.63–5.98)	<0.001	0.0%	0	0.838

^a^For continuous outcomes, the pooled standardized mean difference (SMD) and 95% confidence interval (95%CI). For binary outcomes, the pooled odds ratio (OR) and 95%CI were calculated. I, intervention group; C, control group; TF, trim-and-fill method.

### 3.5 Effects of mind mapping assessed by questionnaire surveys

As shown in Table 2 and Supplementary Figure S5, for binary outcomes, mind mapping outperformed LBL in level of theoretical knowledge, clinical reasoning, learning motivation, autonomous learning ability, problem-solving ability, proficiency in literature retrieval, clinical skills, and course satisfaction (*p* < 0.05). No significant heterogeneity was detected in all meta-analyses with binary outcomes (I^2^ < 50%). Significant publication bias was detected for level of theoretical knowledge, clinical reasoning, learning motivation, autonomous learning ability, and clinical skills (p < 0.05). However, after the trim-and-fill adjustment, the mind mapping still outperformed LBL (p < 0.05) in these 5 meta-analyses with no publication bias (*p* > 0.05).

As shown in Supplementary Table S4, the certainty of the continuous and binary outcomes meta-analyses was rated as very low according to the GRADE assessment.

## 4 Discussion

There has been rapid advancement and continual innovation in the methods of medical education to enhance residents’ engagement, strengthen their theoretical knowledge and develop clinical competency (66). However, traditional LBL remains the dominant approach in China’s medical education, leading to low learning initiation and study outcomes (67). Recently, mind mapping has been widely used in China’s SRT across various clinical specialties. Our systematic review included 52 randomized controlled studies that published from 2017 to 2025, reflecting its rapid adoption as an innovative educational method for residents of SRT. Our meta-analysis identified that mind mapping outperformed LBL in examination scores that comprised theoretical knowledge, case analysis and procedural skills. Questionnaire results further showed that compared with LBL, mind mapping was associated with improvements in level of theoretical knowledge, clinical reasoning, learning motivation, autonomous learning ability, problem-solving ability, proficiency in literature retrieval, clinical skills, teamwork, and course satisfaction. Notably, the effects on clinical reasoning and autonomous learning ability (continuous outcomes) were no longer statistically significant after adjusting for publication bias. To our knowledge, this is the first meta-analysis evaluating the effectiveness of mind mapping compared with LBL in China’s SRT program.

Mind mapping is a visual educational method that promotes the organization, integration and consolidation of knowledge in a diagrammatic way. It was invented in the mid-1970s and developed into its current form by Tony Buzan (7). Early in 2002, Farrand et al. first used mind mapping among medical undergraduates in the United Kingdom (4), in which students received an introduction of mind mapping before the course and were asked to create a mind map afterward. It found that mind mapping could improve long-term factual recall of written information. Subsequent studies showed that mind mapping has been successfully used among medical nursing and pharmacy students in various regions, such as United States (68), Brazil (69) Indonesia (70). Our systematic review identified numerous randomized controlled studies comparing mind mapping with LBL in China’s SRT program. The mind maps included in our systematic review can be categorized into three modes: (1) Faculty-generated mind maps, which were developed and distributed by instructors to provide a structured knowledge scaffold for learning; (2) Student-generated mind maps, which were created by learners independently or guided by the instructor for the concept of mind map; (3) Collaborative co-constructed mind maps, which were initially provided by faculty members as a structured framework and subsequently refined and expanded by students during or after the learning process. Subgroup analyses showed that mind mapping outperformed LBL across most outcomes, except for theoretical knowledge and procedural skill in faculty-generated group, and problem-solving ability in co-constructed group. However, considering the limited number of studies per subgroup (*n* = 2 or 3), further large-scale studies are required to identify the best teaching method of mind mapping in residency education.

Mind mapping was found to enhance memory and comprehension, foster intellectual growth, streamline learning and teaching (6). When creating the mind maps, participants not only actively engage in learning and critical thinking but also improve communication and foster collaboration between students and faculty members (71). The advantages of mind mapping account for its capacity to enhance student examination scores and foster a comprehensive set of competencies, such as knowledge acquisition, self-directed learning and communication skills. On the other hand, it requires training for all students in a class to learn and implement this method, which may be challenging in relatively large groups or be very time-consuming.

While our results demonstrated a statistically improvement in examination scores and competences with mind mapping, interpretation should extend beyond statistical significance to clinical and educational significance. Notably, the observed effect sizes (SMD > 1) are very large in our study, which may imply that mind mapping confers benefits that are both statistically and educationally meaningful. However, these large effect sizes should be viewed with considerable caution, as they are markedly higher than those typically reported in educational interventions. Several factors may account for unusually large effect. First, most included studies had small sample sizes, which tend to generate overestimated and unstable effect estimates, and this imprecision was fully accounted for in our GRADE assessment, thereby contributing to the downgrading of the certainty. Second, the outcomes of the included studies relied heavily on short-term subjective evaluations rather than long-term objective clinical performance assessments, therefore, future data on long-term knowledge retention and actual clinical performance are needed to confirm the real clinical significance. Third, the overall quality of the randomized controlled studies was at high risk of bias, with 98% of studies lacking adequate allocation concealment and blinding and 50% of studies lacking an adequate randomization process, which introduces a substantial risk of selection bias, performance bias and the Hawthorne effect. Residents’ awareness of participating in an educational intervention may have artificially inflated the observed effect sizes. Fourth, the potential publication bias might exaggerate the favorable effects. Therefore, the observed effects may in part reflect systematic bias rather than true intervention superiority and should be interpreted with caution. Despite these uncertainties surrounding the true magnitude of effectiveness, mind mapping remains theoretically plausible and pedagogically valuable as an educational tool.

Notable heterogeneity was observed across the pooled outcomes of examination scores and surveys with continuous outcomes. The heterogeneity remained in most of the meta-analyses after sensitivity analyses and subgroup analyses. Several potential factors might contribute to heterogeneity. First, the substantial variability in the implementation of mind mapping across included studies represents a primary concern to the heterogeneity of this study. Mind mapping was integrated with a range of teaching methods such as PBL, CBL and simulation in 30 of the included studies, and there was a lack of information on faculty training. Moreover, there were missing data regarding to teaching method, such as the frequency of courses, the duration of the intervention, and faculty members matching information, all of which might contribute to the high heterogeneity. Second, diverse medical departments were included in the meta-analysis, which might cause heterogeneity. Our meta-regression analyses suggested that department type could be a potential moderator of the effectiveness of mind mapping, with surgery department tending to show more favorable outcomes in clinical reasoning, learning motivation, and course satisfaction. Third, there were no standardized criteria for the design of examinations or questionnaires. The total score scales used in these assessments differed across the included studies. Many studies did not specify the details of the questionnaire tools, and validation status was rarely reported. These issues may reduce the reliability and comparability of the measured outcomes. However, despite relatively high even relatively high heterogeneity, the random-effect meta-analyses revealed significant results in most meta-analyses that mind mapping outperformed LBL.

The present study has several other limitations. First, the included studies were conducted in China, the generalizability of these findings to other regions remains unclear. Second, despite the use of the PICOS framework to guide the search strategy, the possibility of missing relevant studies cannot be eliminated. Publication bias was also detected in our meta-analyses. After adjustment using the trim-and-fill method, the effect of mind mapping became non-significant in 2 of the meta-analyses, suggesting that our results should be interpreted with caution. Third, while preparing mind mapping courses is time-consuming, none of the included studies assessed faculty satisfaction with this teaching approach. Fourth, the GRADE assessment identified low certainty of evidence for our meta-analyses, further supporting cautious interpretation of the results. Fifth, no included studies assessed cost-effectiveness associated with mind mapping implementation in residency training. Its efficiency, time cost, and scalability in large-scale clinical training programs remain unclear. Overall, future well-designed studies are warranted, which should implement adequate allocation concealment, blinding of outcome assessors, validated outcome measures, long-term follow-up for knowledge retention, assessment of clinical performance outcomes, and evaluation of cost-effectiveness.

## 5 Conclusion

Our meta-analysis indicates that mind mapping may be associated with better examination scores compared with LBL among residents in China’s SRT program. Additionally, mind mapping appeared to be related to favorable changes in the level of theoretical knowledge, clinical reasoning, learning motivation, autonomous learning ability, problem-solving ability, proficiency in literature retrieval, clinical skills, teamwork, and course satisfaction. However, the certainty of evidence was rated as very low, mainly owing to the high risk of bias across included studies (with 98% of studies lacking allocation concealment and blinding), substantial heterogeneity, and publication bias. Some continuous outcomes (clinical reasoning and autonomous learning ability) became non-significant after adjustment for publication bias. Accordingly, the current evidence is insufficient to support strong recommendations for the widespread implementation of mind mapping in medical education. Future well-designed randomized controlled studies with rigorous allocation concealment, blinding of outcome assessors, and validated outcome measures are required to confirm the genuine educational effects of mind mapping.

## Data Availability

The original contributions presented in the study are included in the article/Supplementary material, further inquiries can be directed to the corresponding authors.

## Footnotes

1
http://www.R-project.org

## Supplementary material

Supplementary Figure S1 Forest plots of sensitivity analyses on examination scores in current meta-analyses. The results of sensitivity analyses for the meta-analyses of **(A)** theoretical knowledge scores, **(B)** case analysis scores, **(C)** procedural skill scores, **(D)** level of theoretical knowledge, **(E)** clinical reasoning, **(F)** learning motivation, **(G)** autonomous learning ability, **(H)** problem-solving ability, **(I)** proficiency in literature retrieval, **(J)** clinical skills, **(K)** teamwork, and **(L)** course satisfaction. For each line at the body of the plot, the SMD and 95%CI correspond to the new pooled results after omitting a single study. The large diamond at the bottle of the plot represents the pooled SMD of all studies. The width of the diamond represents with 95%CI.

Supplementary Figure S2 Subgroup meta-analyses of different departments and teaching methods on examination scores results. The meta-analyses on the effect of mind mapping with the subgroup analyses of different departments on **(A)** theoretical knowledge scores, **(B)** case analysis scores, **(C)** procedural skill scores; the meta-analyses on the effect of mind mapping with the subgroup analyses of only mind mapping group and mind mapping combined with other teaching methods on **(D)** theoretical knowledge scores, **(E)** case analysis scores and **(F)** procedural skill scores; the meta-analyses on effect of mind mapping with the subgroup analyses of different methods of generating mind map on the **(G)** theoretical knowledge scores, **(H)** case analysis scores and **(I)** procedural skill scores; the meta-analyses on the effect of mind mapping with the subgroup analyses of different intervention duration on **(J)** theoretical knowledge scores, **(K)** case analysis scores and **(L)** procedural skill scores. The large diamond at the bottle of the plot represents the pooled SMD of all studies. The width of the diamond represents with 95%CI.

Supplementary Figure S3 Forest plots of continuous endpoints of questionnaire surveys results. The meta-analyses on the effect of mind mapping on the level of **(A)** theoretical knowledge, **(B)** clinical reasoning, **(C)** learning motivation, **(D)** autonomous learning ability, **(E)** problem-solving ability, **(F)** proficiency in literature retrieval, **(G)** clinical skills, **(H)** teamwork, and **(I)** course satisfaction. The large diamond at the bottle of the plot represents the pooled SMD of all studies. The width of the diamond represents with 95%CI. The black horizontal bar represents the 95% prediction interval.

Supplementary Figure S4 Subgroup meta-analyses of different departments and teaching methods on questionnaire surveys results. The meta-analyses on the effect of mind mapping with the subgroup analyses of different departments on **(A)** clinical reasoning, **(B)** learning motivation, **(C)** problem-solving ability and **(D)** course satisfaction; The meta-analyses on the effect of mind mapping with the subgroup analyses of only mind mapping group and mind mapping combined with other teaching methods on **(E)** clinical reasoning, **(F)** learning motivation, **(G)** problem-solving ability and **(H)** course satisfaction; The meta-analyses on the effect of mind mapping with the subgroup analyses of different methods of generating mind map on **(I)** clinical reasoning, **(J)** learning motivation, **(K)** problem-solving ability and **(L)** course satisfaction; The meta-analyses on the effect of mind mapping with the subgroup analyses of different intervention duration on **(M)** clinical reasoning, **(N)** learning motivation, **(O)** problem-solving ability and **(P)** course satisfaction. The large diamond at the bottle of the plot represents the pooled SMD of all studies. The width of the diamond represents with 95%CI.

Supplementary Figure S5 Forest plots of binary endpoints of questionnaire surveys results. The meta-analyses on the effect of mind mapping on level of **(A)** theoretical knowledge, **(B)** clinical reasoning, **(C)** learning motivation, **(D)** autonomous learning ability, **(E)** problem-solving ability, **(F)** proficiency in literature retrieval, **(G)** clinical skills and **(H)** course satisfaction. The large diamond at the bottle of the plot represents the pooled OR of all studies. The width of the diamond represents with 95%CI.
