# Supplementary material for: The effectiveness of mind mapping versus lecture-based learning in medical education of China’s standardized residency training: a systematic review and meta-analysis of randomized controlled studies
Source: Front Med (Lausanne). 2026 May 5;13:1789650. doi: 10.3389/fmed.2026.1789650 (PMC13183817; doi:10.3389/fmed.2026.1789650)
Supplement: Supplementary file 2 [file Data_Sheet_2.docx]

**Supplementary Data 2.** Population, intervention, comparison, outcome and study design (PICOS) items and searching strategy in PubMed, China National Knowledge Infrastructure, VIP database and Wanfang database

**Table regarding to PICOS items**

| Population | Postgraduate medical residents |
| --- | --- |
| Intervention | Mind mapping |
| Comparison | Lecture-based learning |
| Outcome | Post learning examinations, such as theoretical knowledge, case analysis and procedural skill; questionnaire surveys such as theoretical knowledge scores, case analysis scores, procedural skill scores, level of theoretical knowledge, clinical reasoning, learning motivation, autonomous learning ability, problem-solving ability, proficiency in literature retrieval, clinical skills, teamwork, and course satisfaction. |
| Study Design | randomized controlled studies |

**Searching strategy in PubMed**

**Complete search strategies:** (((trainee*[tiab] OR resident*[tiab] OR "medical student*"[tiab] OR "internship and residency"[Mesh] OR "students, medical"[Mesh] OR standardised residency training OR residency training OR residency education)) AND ((mind mapping) OR (mind map method) OR (mind map) OR (visual mind mapping) OR (mind mapping teaching))) AND ((learning OR education OR test OR exam OR examination OR student performance OR score*[tiab]))

**Field tags:** The “All Fields” option was used in the search at PubMed.

**Searching strategy in CKNI** **database**

**Complete search strategies:** （主题：规培 + 规范化培训 + 专硕 + 专业硕士 + 专业型硕士）AND（主题：思维导图 + mind mapping + mind map + visual mind mapping + mind mapping teaching + mind map method）AND（主题：学习 + 教育 + 教学 + 考试 + 分数）

**Field tags:** “主题” (Subject).

**Searching strategy in VIP database**

**Complete search strategies:** ((((((题名或关键词=规培 OR 题名或关键词=规范化培训) OR 题名或关键词=专硕) OR 题名或关键词=专业硕士) OR 题名或关键词=专业型硕士) AND (((((题名或关键词=思维导图 OR 题名或关键词=mind mapping) OR 题名或关键词=mind map) OR 题名或关键词=visual mind mapping) OR 题名或关键词=mind mapping teaching) OR 题名或关键词=mind map method)) AND ((((题名或关键词=学习 OR 题名或关键词=教育) OR 题名或关键词=教学) OR 题名或关键词=考试) OR 题名或关键词=分数))

**Field tags:** “题名或关键词” (Title/Keywords).

**Searching strategy in Wanfang** **database**

**Complete search strategies:** (题名或关键词:(规培) or 题名或关键词:(规范化培训) or 题名或关键词:(专硕) or 题名或关键词:(专业硕士) or 题名或关键词:(专业型硕士) or 题名或关键词:(研究生)) AND (题名或关键词:(思维导图) or 题名或关键词:(mind mapping) or 题名或关键词:(mind map) or 题名或关键词:(visual mind mapping) or 题名或关键词:(mind map method) or 题名或关键词:(mind mapping teaching )) AND (题名或关键词:(学习) or 题名或关键词:(教育) or 题名或关键词:(教学) or 题名或关键词:(考试) or 题名或关键词:(分数))

**Field tags:** “题名或关键词” (Title/Keywords).

All search strategies presented above were directly copied from the historical search records of each database (PubMed, CNKI, VIP, and WanFang), ensuring complete consistency with the actual retrieval process performed in this study.
