# Supplementary material for: The effectiveness of mind mapping versus lecture-based learning in medical education of China’s standardized residency training: a systematic review and meta-analysis of randomized controlled studies
Source: Front Med (Lausanne). 2026 May 5;13:1789650. doi: 10.3389/fmed.2026.1789650 (PMC13183817; doi:10.3389/fmed.2026.1789650)
Supplement: Supplementary file 3 [file Table_1.docx]

Supplementary Table 1. Quality assessments of each included study

| First author/Year | Randomization (selection bias) | Alocation concealment (selection bias) | Blinding (participants, performce bias) | Blinding (outcome assessment, detection bias) | Incomplete data report (attrition bias) | Selective data report (reporting bias) | Other bias |
| --- | --- | --- | --- | --- | --- | --- | --- |
| Yuan Cheng/2019 | Low risk | Unclear | Unclear | Unclear | Low risk | Low risk | Unclear |
| Yunfeng Jiang/2024 | Unclear | Unclear | Unclear | Unclear | Low risk | Low risk | Unclear |
| Shanshan Cui/2024 | Unclear | Unclear | Unclear | Unclear | Low risk | Low risk | Unclear |
| Zhenyu Shen/2023 | Unclear | Unclear | Unclear | Unclear | Low risk | Low risk | Unclear |
| Yingbiao Zhu/2023 | Low risk | Unclear | Unclear | Unclear | Low risk | Low risk | Unclear |
| Jiajia Fan/2021 | Low risk | Unclear | Unclear | Unclear | Low risk | Low risk | Unclear |
| Yi Guo/2020 | Unclear | Unclear | Unclear | Unclear | Low risk | Low risk | Unclear |
| Min Lv/2025 | Unclear | Unclear | Unclear | Unclear | Low risk | Low risk | Unclear |
| Jun Liu/2025 | Low risk | Unclear | Unclear | Unclear | Low risk | Low risk | Unclear |
| Dandan Ma/2024 | Low risk | Unclear | Unclear | Unclear | Low risk | Low risk | Unclear |
| Li Qu/2023 | Low risk | Unclear | Unclear | Unclear | Low risk | Low risk | Unclear |
| Hongjuan Shi/2023 | Unclear | Unclear | Unclear | Unclear | Low risk | Low risk | Unclear |
| Liudan Tu/2023 | Low risk | Unclear | Unclear | Unclear | Low risk | Low risk | Unclear |
| Shuo Wu/2022 | Low risk | Unclear | Unclear | Unclear | Low risk | Low risk | Unclear |
| Xin Liao/2022 | Low risk | Unclear | Unclear | Unclear | Low risk | Low risk | Unclear |
| Le Zhang/2021 | Unclear | Unclear | Unclear | Unclear | Low risk | Low risk | Unclear |
| Yan Xu/2018 | Unclear | Unclear | Unclear | Unclear | Low risk | Low risk | Unclear |
| Jing Zhao/2017 | Unclear | Unclear | Unclear | Unclear | Low risk | Low risk | Unclear |
| Jinyu Zhang/2025 | Low risk | Unclear | Unclear | Unclear | Low risk | Low risk | Unclear |
| Ying Zhou/2025 | Unclear | Unclear | Unclear | Unclear | Low risk | Low risk | Unclear |
| Qian Liu/2025 | Low risk | Unclear | Unclear | Unclear | Low risk | Low risk | Unclear |
| Lin Guo/2025 | Unclear | Unclear | Unclear | Unclear | Low risk | Low risk | Unclear |
| Xiaoxue Zhang/2025 | Unclear | Unclear | Unclear | Unclear | Low risk | Low risk | Unclear |
| Yue Yu/2024 | Low risk | Unclear | Unclear | Unclear | Low risk | Low risk | Unclear |
| Ning Du/2024 | Low risk | Unclear | Unclear | Unclear | Low risk | Low risk | Unclear |
| Yanming Zhang/2024 | Unclear | Unclear | Unclear | Unclear | Low risk | Low risk | Unclear |
| Jia Chen/2024 | Unclear | Unclear | Unclear | Unclear | Low risk | Low risk | Unclear |
| Yipeng Liu/2024 | Unclear | Unclear | Unclear | Unclear | Low risk | Low risk | Unclear |
| Min Tang/2024 | Unclear | Unclear | Unclear | Unclear | Low risk | Low risk | Unclear |
| Jin Zhang/2024 | Low risk | Unclear | Unclear | Unclear | Low risk | Low risk | Unclear |
| Yanqian Deng/2024 | Low risk | Unclear | Unclear | Unclear | Low risk | Low risk | Unclear |
| Wenjie Xu/2024 | Low risk | Unclear | Unclear | Unclear | Low risk | Low risk | Unclear |
| Jingmin Dong/2023 | Unclear | Unclear | Unclear | Unclear | Low risk | Low risk | Unclear |
| Lingtao Liu/2023 | Unclear | Unclear | Unclear | Unclear | Low risk | Low risk | Unclear |
| Fan Liu/2023 | Unclear | Unclear | Unclear | Unclear | Low risk | Low risk | Unclear |
| Shengqun Jiang/2022 | Low risk | Unclear | Unclear | Unclear | Low risk | Low risk | Unclear |
| Yafang Wei/2022 | Unclear | Unclear | Unclear | Unclear | Low risk | Low risk | Unclear |
| Hongyan Li/2022 | Unclear | Unclear | Unclear | Unclear | Low risk | Low risk | Unclear |
| Bing Zhou/2021 | Low risk | Unclear | Unclear | Unclear | Low risk | Low risk | Unclear |
| Shuai Fu/2021 | Unclear | Unclear | Unclear | Unclear | Low risk | Low risk | Unclear |
| Li Yao/2019 | Unclear | Unclear | Unclear | Unclear | Low risk | Low risk | Unclear |
| Zhouwei Xu/2025 | Low risk | Low risk | Low risk | Low risk | Low risk | Low risk | Low risk |
| Rong Liu/2025 | Unclear | Unclear | Unclear | Unclear | Low risk | Low risk | Unclear |
| Yao Hu/2025 | Low risk | Unclear | Unclear | Unclear | Low risk | Low risk | Unclear |
| Jianping Gong/2023 | Low risk | Unclear | Unclear | Unclear | Low risk | Low risk | Unclear |
| Wenjuan Wang/2020 | Low risk | Unclear | Unclear | Unclear | Low risk | Low risk | Unclear |
| Shushu Zhang/2024 | Low risk | Unclear | Unclear | Unclear | Low risk | Low risk | Unclear |
| Dongzhu Zhang/2019 | Unclear | Unclear | Unclear | Unclear | Low risk | Low risk | Unclear |
| Yuehua Pu/2017 | Low risk | Unclear | Unclear | Unclear | Low risk | Low risk | Unclear |
| Jun Li/2023 | Unclear | Unclear | Unclear | Unclear | Low risk | Low risk | Unclear |
| Qingsong Zhang/2025 | Low risk | Unclear | Unclear | Unclear | Low risk | Low risk | Unclear |
| Yuanhui Sun/2025 | Low risk | Unclear | Unclear | Unclear | Low risk | Low risk | Unclear |
