# Supplementary material for: The effectiveness of mind mapping versus lecture-based learning in medical education of China’s standardized residency training: a systematic review and meta-analysis of randomized controlled studies
Source: Front Med (Lausanne). 2026 May 5;13:1789650. doi: 10.3389/fmed.2026.1789650 (PMC13183817; doi:10.3389/fmed.2026.1789650)
Supplement: Supplementary file 4 [file Table_2.docx]

**Supplementary Table 2.** The sensitivity analyses of current meta-analyses

| Omitted study | SMD (95%CI) after omitting study | I2 |
| --- | --- | --- |
| **Theoretical knowledge scores** |  |  |
| Omitting Shanshan Cui/2024 | 1.47 (1.18-1.77) | 87.9% |
| Omitting Zhenyu Shen/2023 | 1.48 (1.19-1.77) | 87.8% |
| Omitting Yingbiao Zhu/2023 | 1.48 (1.19-1.77) | 86.7% |
| Omitting Jiajia Fan/2021 | 1.45 (1.16-1.75) | 88.0% |
| Omitting Yi Guo/2020 | 1.45 (1.15-1.75) | 88.0% |
| Omitting Min Lv/2025 | 1.47 (1.17-1.76) | 88.0% |
| Omitting Jun Liu/2025 | 1.44 (1.14-1.73) | 87.6% |
| Omitting Dandan Ma/2024 | 1.47 (1.17-1.77) | 87.8% |
| Omitting Li Qu/2023 | 1.44 (1.15-1.74) | 87.9% |
| Omitting Hongjuan Shi/2023 | 1.47 (1.18-1.77) | 87.9% |
| Omitting Liudan Tu/2023 | 1.44 (1.15-1.74) | 87.9% |
| Omitting Xin Liao/2022 | 1.43 (1.14-1.72) | 87.4% |
| Omitting Le Zhang/2021 | 1.46 (1.16-1.76) | 88.0% |
| Omitting Yan Xu/2018 | 1.46 (1.17-1.76) | 88.0% |
| Omitting Jing Zhao/2017 | 1.48 (1.18-1.77) | 87.8% |
| Omitting Jinyu Zhang/2025 | 1.47 (1.17-1.77) | 87.9% |
| Omitting Ying Zhou/2025 | 1.46 (1.17-1.76) | 88.0% |
| Omitting Qian Liu/2025 | 1.44 (1.14-1.73) | 87.8% |
| Omitting Lin Guo/2025 | 1.48 (1.18-1.77) | 87.8% |
| Omitting Yue Yu/2024 | 1.47 (1.18-1.77) | 87.9% |
| Omitting Yanming Zhang/2024 | 1.44 (1.15-1.74) | 87.8% |
| Omitting Jia Chen/2024 | 1.46 (1.16-1.76) | 88.0% |
| Omitting Yipeng Liu/2024 | 1.46 (1.16-1.76) | 88.0% |
| Omitting Min Tang/2024 | 1.46 (1.17-1.76) | 88.0% |
| Omitting Jin Zhang/2024 | 1.46 (1.16-1.75) | 88.0% |
| Omitting Wenjie Xu/2024 | 1.37 (1.12-1.63) | 85.9% |
| Omitting Jingmin Dong/2023 | 1.45 (1.15-1.74) | 87.9% |
| Omitting Fan Liu/2023 | 1.46 (1.16-1.75) | 88.0% |
| Omitting Shengqun Jiang/2022 | 1.47 (1.18-1.77) | 87.9% |
| Omitting Yafang Wei/2022 | 1.39 (1.12-1.67) | 86.1% |
| Omitting Hongyan Li/2022 | 1.47 (1.17-1.77) | 88.0% |
| Omitting Bing Zhou/2021 | 1.48 (1.18-1.77) | 87.7% |
| Omitting Shuai Fu/2021 | 1.46 (1.16-1.76) | 88.0% |
| Omitting Li Yao/2019 | 1.47 (1.17-1.76) | 88.0% |
| Omitting Rong Liu/2025 | 1.44 (1.15-1.74) | 87.9% |
| Omitting Yao Hu/2025 | 1.48 (1.18-1.77) | 87.8% |
| Omitting Jianping Gong/2023 | 1.41 (1.13-1.69) | 87.0% |
| Omitting Wenjuan Wang/2020 | 1.43 (1.14-1.72) | 87.5% |
| Omitting Shushu Zhang/2024 | 1.42 (1.13-1.71) | 87.0% |
| Omitting Dongzhu Zhang/2019 | 1.45 (1.16-1.75) | 88.0% |
| Omitting Jun Li/2023 | 1.41 (1.12-1.69) | 86.9% |
| Omitting studies with high/unclear randomization bias | 1.66 (1.16-2.17) | 87.7% |
| **Case analysis scores** |  |  |
| Omitting Jun Liu/2025 | 1.38 (0.97-1.79) | 83.1% |
| Omitting Hongjuan Shi/2023 | 1.37 (0.95-1.78) | 83.5% |
| Omitting Shuo Wu/2022 | 1.17 (0.89-1.45) | 73.7% |
| Omitting Jinyu Zhang/2025 | 1.35 (0.94-1.77) | 83.5% |
| Omitting Ying Zhou/2025 | 1.35 (0.94-1.77) | 83.6% |
| Omitting Qian Liu/2025 | 1.31 (0.9-1.72) | 82.7% |
| Omitting Lin Guo/2025 | 1.38 (0.98-1.79) | 83.1% |
| Omitting Xiaoxue Zhang/2025 | 1.29 (0.89-1.68) | 82.2% |
| Omitting Yanming Zhang/2024 | 1.35 (0.93-1.77) | 83.5% |
| Omitting Jia Chen/2024 | 1.36 (0.95-1.77) | 83.6% |
| Omitting Yanqian Deng/2024 | 1.36 (0.94-1.77) | 83.6% |
| Omitting Lingtao Liu/2023 | 1.34 (0.93-1.75) | 83.5% |
| Omitting Bing Zhou/2021 | 1.39 (0.99-1.79) | 82.1% |
| Omitting Shuai Fu/2021 | 1.37 (0.96-1.78) | 83.5% |
| Omitting Li Yao/2019 | 1.4 (1.01-1.79) | 80.4% |
| Omitting Wenjuan Wang/2020 | 1.27 (0.89-1.65) | 79.7% |
| Omitting Qingsong Zhang/2025 | 1.31 (0.9-1.71) | 82.7% |
| Omitting studies with high/unclear randomization bias | 1.67 (0.91-2.44) | 82.5% |
| **Procedural skill scores** |  |  |
| Omitting Yuan Cheng/2019 | 1.71 (1.25 to 2.18) | 90.3% |
| Omitting Shanshan Cui/2024 | 1.73 (1.27 to 2.19) | 90.2% |
| Omitting Jiajia Fan/2021 | 1.69 (1.22 to 2.16) | 90.4% |
| Omitting Jun Liu/2025 | 1.66 (1.19 to 2.13) | 90.0% |
| Omitting Li Qu/2023 | 1.73 (1.27 to 2.19) | 90.1% |
| Omitting Hongjuan Shi/2023 | 1.71 (1.25 to 2.18) | 90.4% |
| Omitting Xin Liao/2022 | 1.64 (1.18 to 2.1) | 89.6% |
| Omitting Le Zhang/2021 | 1.7 (1.23 to 2.17) | 90.5% |
| Omitting Yan Xu/2018 | 1.66 (1.19 to 2.12) | 90.3% |
| Omitting Jing Zhao/2017 | 1.7 (1.23 to 2.17) | 90.5% |
| Omitting Lin Guo/2025 | 1.7 (1.23 to 2.17) | 90.4% |
| Omitting Xiaoxue Zhang/2025 | 1.67 (1.2 to 2.14) | 90.3% |
| Omitting Yue Yu/2024 | 1.72 (1.26 to 2.19) | 90.2% |
| Omitting Ning Du/2024 | 1.7 (1.23 to 2.17) | 90.4% |
| Omitting Min Tang/2024 | 1.72 (1.25 to 2.18) | 90.4% |
| Omitting Jin Zhang/2024 | 1.7 (1.23 to 2.17) | 90.5% |
| Omitting Wenjie Xu/2024 | 1.55 (1.16 to 1.95) | 88.2% |
| Omitting Jingmin Dong/2023 | 1.71 (1.24 to 2.18) | 90.4% |
| Omitting Shengqun Jiang/2022 | 1.71 (1.24 to 2.18) | 90.4% |
| Omitting Bing Zhou/2021 | 1.73 (1.27 to 2.19) | 89.9% |
| Omitting Li Yao/2019 | 1.73 (1.27 to 2.19) | 89.7% |
| Omitting Rong Liu/2025 | 1.68 (1.21 to 2.15) | 90.4% |
| Omitting Yao Hu/2025 | 1.71 (1.24 to 2.18) | 90.4% |
| Omitting Jianping Gong/2023 | 1.59 (1.16 to 2.02) | 88.9% |
| Omitting Wenjuan Wang/2020 | 1.66 (1.19 to 2.13) | 90.0% |
| Omitting Dongzhu Zhang/2019 | 1.7 (1.23 to 2.16) | 90.5% |
| Omitting Jun Li/2023 | 1.6 (1.16 to 2.04) | 89.1% |
| Omitting Yuanhui Sun/2025 | 1.59 (1.16 to 2.02) | 89.3% |
| Omitting high/unclear randomization bias studies | 1.92 (1.19-2.66) | 90.1% |
| **Level of theoretical knowledge** |  |  |
| Omitting Yunfeng Jiang/2024 | 1.1 (0.56-1.64) | 82.4% |
| Omitting Hongjuan Shi/2023 | 1.17 (0.66-1.69) | 81.4% |
| Omitting Xin Liao/2022 | 0.99 (0.53-1.45) | 75.6% |
| Omitting Jing Zhao/2017 | 1.14 (0.6-1.68) | 82.7% |
| Omitting Qian Liu/2025 | 1.06 (0.54-1.59) | 81.8% |
| Omitting Min Tang/2024 | 1.15 (0.62-1.68) | 82.5% |
| Omitting Zhouwei Xu/2025 | 1.13 (0.58-1.68) | 82.7% |
| Omitting Wenjuan Wang/2020 | 1.2 (0.76-1.64) | 74.9% |
| Omitting Yuehua Pu/2017 | 0.93 (0.57-1.28) | 74.3% |
| Omitting studies with high/unclear randomization bias | 1.4 (0.5-2.3) | 80.3% |
| **Clinical reasoning** |  |  |
| Omitting Le Zhang/2021 | 2.03 (0.88-3.17) | 90.8% |
| Omitting Jinyu Zhang/2025 | 2.08 (0.95-3.21) | 90.4% |
| Omitting Yue Yu/2024 | 2.04 (0.9-3.19) | 90.8% |
| Omitting Min Tang/2024 | 2.07 (0.94-3.2) | 90.6% |
| Omitting Jin Zhang/2024 | 2 (0.84-3.15) | 90.6% |
| Omitting Yanqian Deng/2024 | 2.05 (0.91-3.19) | 90.7% |
| Omitting Shengqun Jiang/2022 | 1.4 (0.96-1.85) | 81.6% |
| Omitting Zhouwei Xu/2025 | 2.07 (0.94-3.2) | 89.9% |
| Omitting Rong Liu/2025 | 2.01 (0.85-3.16) | 90.7% |
| Omitting Yao Hu/2025 | 2.06 (0.92-3.2) | 90.7% |
| Omitting Jianping Gong/2023 | 1.93 (0.78-3.09) | 89.7% |
| Omitting Wenjuan Wang/2020 | 1.95 (0.8-3.11) | 89.9% |
| Omitting Yuehua Pu/2017 | 1.77 (0.73-2.81) | 89.2% |
| Omitting studies with high/unclear randomization bias | 2.23 (0.84-3.63) | 90.0% |
| **Learning motivation** |  |  |
| Omitting Yi Guo/2020 | 1.87 (1.02-2.71) | 87.9% |
| Omitting Min Lv/2025 | 1.82 (0.96-2.67) | 88.0% |
| Omitting Xin Liao/2022 | 1.74 (0.91-2.58) | 85.8% |
| Omitting Le Zhang/2021 | 1.86 (1.01-2.71) | 88.0% |
| Omitting Jing Zhao/2017 | 1.82 (0.96-2.68) | 88.0% |
| Omitting Ying Zhou/2025 | 1.89 (1.05-2.72) | 87.5% |
| Omitting Qian Liu/2025 | 1.87 (1.02-2.72) | 87.8% |
| Omitting Yue Yu/2024 | 1.84 (0.98-2.7) | 88.0% |
| Omitting Min Tang/2024 | 1.88 (1.04-2.72) | 87.5% |
| Omitting Jin Zhang/2024 | 1.86 (1.01-2.71) | 87.9% |
| Omitting Yanqian Deng/2024 | 1.88 (1.03-2.72) | 87.3% |
| Omitting Shengqun Jiang/2022 | 1.44 (1.11-1.78) | 75.5% |
| Omitting Zhouwei Xu/2025 | 1.84 (0.98-2.7) | 88.0% |
| Omitting Rong Liu/2025 | 1.85 (1-2.7) | 88.0% |
| Omitting Wenjuan Wang/2020 | 1.73 (0.9-2.56) | 85.6% |
| Omitting studies with high/unclear randomization bias | 2.41 (0.83-4) | 87.1% |
| **Autonomous learning ability** |  |  |
| Omitting Yi Guo/2020 | 1.77 (0.34-3.21) | 90.2% |
| Omitting Jun Liu/2025 | 2 (0.6-3.41) | 89.8% |
| Omitting Hongjuan Shi/2023 | 1.99 (0.58-3.4) | 90.3% |
| Omitting Le Zhang/2021 | 1.88 (0.43-3.33) | 90.8% |
| Omitting Jing Zhao/2017 | 1.96 (0.52-3.39) | 90.8% |
| Omitting Xiaoxue Zhang/2025 | 1.86 (0.41-3.32) | 90.6% |
| Omitting Jin Zhang/2024 | 1.89 (0.43-3.34) | 90.6% |
| Omitting Yanqian Deng/2024 | 1.94 (0.5-3.38) | 90.8% |
| Omitting Shengqun Jiang/2022 | 1.1 (0.75-1.46) | 62.7% |
| Omitting studies with high/unclear randomization bias | 2.52 (-0.56-5.6) | 89.6% |
| **Problem solving ability** |  |  |
| Omitting Yi Guo/2020 | 2.27 (1.01-3.52) | 89.4% |
| Omitting Min Lv/2025 | 2.43 (1.17-3.69) | 89.9% |
| Omitting Hongjuan Shi/2023 | 2.48 (1.24-3.71) | 88.9% |
| Omitting Xin Liao/2022 | 2.38 (1.11-3.66) | 89.9% |
| Omitting Qian Liu/2025 | 2.45 (1.21-3.7) | 89.6% |
| Omitting Yue Yu/2024 | 2.44 (1.19-3.7) | 89.8% |
| Omitting Jin Zhang/2024 | 2.4 (1.13-3.67) | 89.9% |
| Omitting Shengqun Jiang/2022 | 1.75 (1.24-2.26) | 75.9% |
| Omitting Rong Liu/2025 | 2.45 (1.21-3.7) | 89.7% |
| Omitting Qingsong Zhang/2025 | 2.39 (1.12-3.66) | 89.9% |
| Omitting Yuanhui Sun/2025 | 2.17 (0.97-3.38) | 87.1% |
| Omitting studies with high/unclear randomization bias | 2.78 (1.01-4.56) | 88.8% |
| **Proficiency in literature retrieval** |  |  |
| Omitting Yi Guo/2020 | 0.99 (0.67-1.31) | 0.0% |
| Omitting Hongjuan Shi/2023 | 1.48 (0.63-2.34) | 67.1% |
| Omitting Qian Liu/2025 | 1.43 (0.45-2.41) | 76.0% |
| Omitting Jin Zhang/2024 | 1.33 (0.29-2.38) | 75.3% |
| Omitting studies with high/unclear randomization bias | 1.13 (0.73-1.53) | 65.1% |
| **Teamwork** |  |  |
| Omitting Hongjuan Shi/2023 | 2.39 (0.18-4.59) | 94.7% |
| Omitting Le Zhang/2021 | 2.3 (0.06-4.54) | 95.3% |
| Omitting Qian Liu/2025 | 2.28 (0.02-4.53) | 95.3% |
| Omitting Min Tang/2024 | 2.44 (0.27-4.61) | 94.1% |
| Omitting Shengqun Jiang/2022 | 1.25 (0.73-1.76) | 91.0% |
| Omitting Zhouwei Xu/2025 | 2.17 (-0.1-4.44) | 92.6% |
| Omitting Yao Hu/2025 | 2.38 (0.17-4.59) | 94.7% |
| Omitting studies with high/unclear randomization bias | 3.15 (-0.13-6.43) | 94.4% |
| **Clinical skills** |  |  |
| Omitting Yunfeng Jiang/2024 | 1.55 (0.58-2.51) | 91.8% |
| Omitting Le Zhang/2021 | 1.57 (0.62-2.51) | 91.8% |
| Omitting Yue Yu/2024 | 1.41 (0.46-2.36) | 91.1% |
| Omitting Rong Liu/2025 | 1.43 (0.47-2.38) | 91.4% |
| Omitting Yao Hu/2025 | 1.77 (1.12-2.42) | 76.4% |
| Omitting Qingsong Zhang/2025 | 1.47 (0.51-2.44) | 91.7% |
| Omitting Yuanhui Sun/2025 | 1.17 (0.51-1.84) | 86.6% |
| Omitting studies with high/unclear randomization bias | 1.63 (0.16-3.11) | 90.2% |
| **Course satisfaction** |  |  |
| Omitting Yi Guo/2020 | 2.04 (0.89-3.2) | 90.9% |
| Omitting Le Zhang/2021 | 2 (0.83-3.17) | 91.0% |
| Omitting Ying Zhou/2025 | 2.06 (0.91-3.21) | 90.6% |
| Omitting Xiaoxue Zhang/2025 | 1.94 (0.76-3.11) | 90.8% |
| Omitting Yue Yu/2024 | 2.01 (0.84-3.18) | 91.0% |
| Omitting Ning Du/2024 | 2.03 (0.87-3.2) | 90.9% |
| Omitting Min Tang/2024 | 2.05 (0.9-3.21) | 90.6% |
| Omitting Shengqun Jiang/2022 | 1.42 (0.9-1.94) | 81.8% |
| Omitting Zhouwei Xu/2025 | 2 (0.82-3.17) | 91.0% |
| Omitting Wenjuan Wang/2020 | 2.07 (0.92-3.21) | 90.1% |
| Omitting Yuehua Pu/2017 | 1.83 (0.69-2.97) | 90.2% |
| Omitting Jun Li/2023 | 1.82 (0.68-2.95) | 88.4% |
| Omitting studies with high/unclear randomization bias | 2.49 (0.35-4.64) | 90.1% |

the pooled standardized mean difference (SMD), 95%confidence interval (95%CI) and I square (I^2^) after omitting relevant study were calculated.
