# Supplementary material for: The effectiveness of mind mapping versus lecture-based learning in medical education of China’s standardized residency training: a systematic review and meta-analysis of randomized controlled studies
Source: Front Med (Lausanne). 2026 May 5;13:1789650. doi: 10.3389/fmed.2026.1789650 (PMC13183817; doi:10.3389/fmed.2026.1789650)
Supplement: Supplementary file 5 [file Table_3.docx]

**Supplementary Table 3.** Meta-regression results for heterogeneity moderators

| Item | Moderators | Subgroup | Meta-regression coefficient (95% CI) | p value | R^2^ |
| --- | --- | --- | --- | --- | --- |
| Theoretical knowledge scores | Department | Anesthesiology | ref | ref | 0.51% |
|  |  | Diagnostic imaging | -0.19 (-1.46 to 1.08) | 0.771 |  |
|  |  | Internal Medicine | 0.02 (-1.13 to 1.18) | 0.969 |  |
|  |  | Not specified | -0.05 (-1.73 to 1.64) | 0.955 |  |
|  |  | Surgery | -0.89 (-2.21 to 0.42) | 0.184 |  |
|  | Interventions | Mind map with other methods | ref | ref | 0.92% |
|  |  | Only mind map | -0.37 (-0.98 to 0.23) | 0.229 |  |
|  | Mind map methods | Co-generated | ref | ref | 0% |
|  |  | Faculty-generated | 0.45 (-0.83 to 1.72) | 0.493 |  |
|  |  | Student-generated | 0.13 (-0.57 to 0.82) | 0.721 |  |
|  | Intervention duration | Equal or over 3 months | ref | ref | 1.51% |
|  |  | Less than 3 months | -0.16 (-1.28 to 0.97) | 0.787 |  |
|  |  | Not reported | 0.41 (-0.60 to 1.41) | 0.429 |  |
| Case analysis scores | Department | Diagnostic imaging | ref | ref | 0% |
|  |  | Internal Medicine | 0.51 (-0.79 to 1.82) | 0.44 |  |
|  |  | Surgery | 1.10 (-0.37 to 2.57) | 0.144 |  |
|  | Interventions | Mind map with other methods | ref | ref | 0% |
|  |  | Only mind map | 0.36 (-0.51 to 1.24) | 0.417 |  |
|  | Mind map methods | Co-generated | ref | ref | 5.29% |
|  |  | Student-generated | 0.56 (-0.31 to 1.43) | 0.204 |  |
|  | Intervention duration | Equal or over 3 months | ref | ref | 1.15% |
|  |  | Less than 3 months | -0.95 (-2.5 to 0.60) | 0.228 |  |
|  |  | Not reported | -0.92 (-2.05 to 0.22) | 0.114 |  |
| Procedural skill scores | Department | Anesthesiology | ref | ref | 0% |
|  |  | Diagnostic imaging | 0 (-1.59 to 1.59) | 0.996 |  |
|  |  | Internal Medicine | 0.19 (-1.24 to 1.62) | 0.797 |  |
|  |  | Not specified | -0.4 (-3.21 to 2.4) | 0.778 |  |
|  |  | Surgery | -0.92 (-3.08 to 1.25) | 0.406 |  |
|  | Interventions | Mind map with other methods | ref | ref | 0.29% |
|  |  | Only mind map | -0.56 (-1.52 to 0.41) | 0.258 |  |
|  | Mind map methods | Co-generated | ref | ref | 4.34% |
|  |  | Faculty-generated | 1.78 (-0.07 to 3.63) | 0.059 |  |
|  |  | Student-generated | 0.37 (-0.58 to 1.31) | 0.447 |  |
|  | Intervention duration | Equal or over 3 months | ref | ref | 0% |
|  |  | Less than 3 months | -0.32 (-3.02 to 2.38) | 0.816 |  |
|  |  | Not reported | 0.21 (-2.31 to 2.74) | 0.869 |  |
| Clinical reasoning | Department | Anesthesiology | ref | ref | **83.95%** |
|  |  | Diagnostic imaging | -0.79 (-2.85 to 1.27) | 0.452 |  |
|  |  | Internal Medicine | -1.05 (-2.83 to 0.74) | 0.252 |  |
|  |  | Surgery | **6.23 (3.27 to 9.19)** | **<0.001** |  |
|  | Interventions | Mind map with other methods | ref | ref | 0% |
|  |  | Only mind map | 0.58 (-2.68 to 3.85) | 0.727 |  |
|  | Mind map methods | Co-generated | ref | ref | 0% |
|  |  | Student-generated | 1.43 (-2.82 to 5.67) | 0.51 |  |
|  | Intervention duration | Less than 3 months | ref | ref | 0% |
|  |  | Not reported | 1.25 (-1.09 to 3.59) | 0.294 |  |
| Problem solving ability | Department | Diagnostic imaging | ref | ref | 0% |
|  |  | Internal Medicine | 0.20 (-2.93 to 3.34) | 0.899 |  |
|  |  | Surgery | 2.07 (-1.32 to 5.46) | 0.232 |  |
|  | Interventions | Mind map with other methods | ref | ref | 0% |
|  |  | Only mind map | -1.37 (-3.94 to 1.19) | 0.294 |  |
|  | Mind map methods | Co-generated | ref | ref | 0% |
|  |  | Student-generated | 1.49 (-2.82 to 5.80) | 0.498 |  |
|  | Intervention duration | Less than 3 months | ref | ref | 0% |
|  |  | Not reported | 1.04 (-2.23 to 4.31) | 0.533 |  |
| Learning motivation | Department | Diagnostic imaging | ref | ref | **16.67%** |
|  |  | Internal Medicine | -0.35 (-2.43 to 1.72) | 0.74 |  |
|  |  | Not specified | -0.02 (-3.49 to 3.45) | 0.99 |  |
|  |  | Surgery | **2.95 (0.08 to 5.82)** | **0.044** |  |
|  | Interventions | Mind map with other methods | ref | ref | 0% |
|  |  | Only mind map | -0.11 (-2.81 to 2.59) | 0.936 |  |
|  | Mind map methods | Co-generated | ref | ref | 0% |
|  |  | Faculty-generated | 0.84 (-4.33 to 6.02) | 0.75 |  |
|  |  | Student-generated | 1.23 (-2.59 to 5.06) | 0.527 |  |
|  | Intervention duration | Less than 3 months | ref | ref | 4.97% |
|  |  | Not reported | 1.26 (-0.58 to 3.09) | 0.18 |  |
| Course satisfaction | Department | Anesthesiology | ref | ref | **78.03%** |
|  |  | Internal Medicine | 0.55 (-1.39 to 2.49) | 0.579 |  |
|  |  | Surgery | **7.08 (3.98 to 10.17)** | **<0.001** |  |
|  | Interventions | Mind map with other methods | ref | ref | 0% |
|  |  | Only mind map | -0.01 (-2.78 to 2.76) | 0.995 |  |
|  | Mind map methods | Co-generated | ref | ref | 0% |
|  |  | Student-generated | 1.10 (-1.55 to 3.76) | 0.416 |  |
|  | Intervention duration | Less than 3 months | ref | ref | 0% |
|  |  | Not reported | 0.68 (-1.85 to 3.20) | 0.599 |  |
