# Supplementary material for: The effectiveness of mind mapping versus lecture-based learning in medical education of China’s standardized residency training: a systematic review and meta-analysis of randomized controlled studies
Source: Front Med (Lausanne). 2026 May 5;13:1789650. doi: 10.3389/fmed.2026.1789650 (PMC13183817; doi:10.3389/fmed.2026.1789650)
Supplement: Supplementary file 6 [file Table_4.docx]

**Supplementary Table 4.** GRADE assessment for certainty of evidence in current meta-analysis

| Variable outcomes | Risk of bias^a^ | Inconsistency^b^ | Indirectness | Imprecision^c^ | Publication bias^d^ | Quality of evidence |
| --- | --- | --- | --- | --- | --- | --- |
| **Continuous outcomes** |  |  |  |  |  |  |
| Theoretical knowledge scores | Very Serious (-2) | Serious (-1) | No | Serious (-1) | Serious (-1) | Very low certainty |
| Case analysis scores | Very Serious (-2) | Serious (-1) | No | Serious (-1) | Serious (-1) | Very low certainty |
| Procedural skill scores | Very Serious (-2) | Serious (-1) | No | Serious (-1) | Serious (-1) | Very low certainty |
| Level of theoretical knowledge | Very Serious (-2) | Serious (-1) | No | Serious (-1) | No | Very low certainty |
| Clinical reasoning | Very Serious (-2) | Serious (-1) | No | Serious (-1) | Serious (-1) | Very low certainty |
| Learning motivation | Very Serious (-2) | Serious (-1) | No | Serious (-1) | No | Very low certainty |
| Autonomous learning ability | Very Serious (-2) | Serious (-1) | No | Serious (-1) | Serious (-1) | Very low certainty |
| Problem-solving ability | Very Serious (-2) | Serious (-1) | No | Serious (-1) | Serious (-1) | Very low certainty |
| Proficiency in literature retrieval | Very Serious (-2) | Serious (-1) | No | Serious (-1) | No | Very low certainty |
| Clinical skills | Very Serious (-2) | Serious (-1) | No | Serious (-1) | Serious (-1) | Very low certainty |
| Teamwork | Very Serious (-2) | Serious (-1) | No | Serious (-1) | No | Very low certainty |
| Course satisfaction | Very Serious (-2) | Serious (-1) | No | Serious (-1) | No | Very low certainty |
| **Binary outcomes** |  |  |  |  |  |  |
| Level of theoretical knowledge | Very Serious (-2) | No | No | Serious (-1) | Serious (-1) | Very low certainty |
| Clinical reasoning | Very Serious (-2) | No | No | Serious (-1) | Serious (-1) | Very low certainty |
| Learning motivation | Very Serious (-2) | No | No | Serious (-1) | Serious (-1) | Very low certainty |
| Autonomous learning ability | Very Serious (-2) | No | No | Serious (-1) | Serious (-1) | Very low certainty |
| Problem solving ability | Very Serious (-2) | No | No | Serious (-1) | No | Very low certainty |
| Proficiency in literature retrieval | Very Serious (-2) | No | No | Serious (-1) | No | Very low certainty |
| Clinical skills | Very Serious (-2) | No | No | Serious (-1) | No | Very low certainty |
| Course satisfaction | Very Serious (-2) | No | No | Serious (-1) | Serious (-1) | Very low certainty |

^a^Downgraded two level for significant concerns about lack of randomization, allocation concealment, and blinding process in a substantial proportion of studies in our meta-analyses; ^b^Downgraded one level for the significant heterogeneity (I^2^>50%) in relative meta-analysis; ^c^Downgraded one level for the small sample size of the included randomized controlled studies; ^d^Downgraded one level for the significant publication bias.
