# Supplementary material for: The effectiveness of mind mapping versus lecture-based learning in medical education of China’s standardized residency training: a systematic review and meta-analysis of randomized controlled studies
Source: Front Med (Lausanne). 2026 May 5;13:1789650. doi: 10.3389/fmed.2026.1789650 (PMC13183817; doi:10.3389/fmed.2026.1789650)
Supplement: Supplementary Figure S1 — Forest plots of sensitivity analyses on examination scores in current meta-analyses. The results of sensitivity analyses for the meta-analyses of (A) theoretical knowledge scores, (B) case analysis scores, (C) procedural skill scores, (D) level of theoretical knowledge, (E) clinical reasoning, (F) learning motivation, (G) autonomous learning ability, (H) problem-solving ability, (I) proficiency in literature retrieval, (J) clinical skills, (K) teamwork, and (L) course satisfaction. For each line at the body of the plot, the SMD and 95%CI correspond to the new pooled results after omitting a single study. The large diamond at the bottle of the plot represents the pooled SMD of all studies. The width of the diamond represents with 95%CI. [file Image_1.pdf]

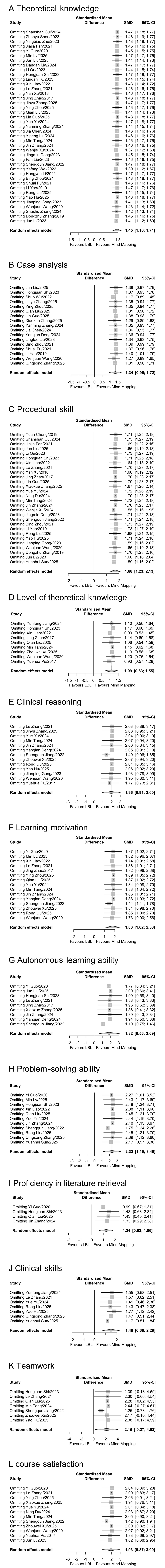
A Theoretical knowledge
Study
Standardised Mean Difference
SMD
95%-CI
Omitting Shanshan Cui/2024 1.47 [1.18; 1.77]
Omitting Zhenyu Shen/2023 1.48 [1.19; 1.77]
Omitting Yingbiao Zhu/2023 1.48 [1.19; 1.77]
Omitting Jiajia Fan/2021 1.45 [1.16; 1.75]
Omitting Yi Guo/2020 1.45 [1.15; 1.75]
Omitting Min Lv/2025 1.47 [1.17; 1.76]
Omitting Jun Liu/2025 1.44 [1.14; 1.73]
Omitting Dandan Ma/2024 1.47 [1.17; 1.77]
Omitting Li Qu/2023 1.44 [1.15; 1.74]
Omitting Hongjuan Shi/2023 1.47 [1.18; 1.77]
Omitting Liudan Tu/2023 1.44 [1.15; 1.74]
Omitting Xin Liao/2022 1.43 [1.14; 1.72]
Omitting Le Zhang/2021 1.46 [1.16; 1.76]
Omitting Yan Xu/2018 1.46 [1.17; 1.76]
Omitting Jing Zhao/2017 1.48 [1.18; 1.77]
Omitting Jinyu Zhang/2025 1.47 [1.17; 1.77]
Omitting Ying Zhou/2025 1.46 [1.17; 1.76]
Omitting Qian Liu/2025 1.44 [1.14; 1.73]
Omitting Lin Guo/2025 1.48 [1.18; 1.77]
Omitting Yue Yu/2024 1.47 [1.18; 1.77]
Omitting Yanming Zhang/2024 1.44 [1.15; 1.74]
Omitting Jia Chen/2024 1.46 [1.16; 1.76]
Omitting Yipeng Liu/2024 1.46 [1.16; 1.76]
Omitting Min Tang/2024 1.46 [1.17; 1.76]
Omitting Jin Zhang/2024 1.46 [1.16; 1.75]
Omitting Wenjie Xu/2024 1.37 [1.12; 1.63]
Omitting Jingmin Dong/2023 1.45 [1.15; 1.74]
Omitting Fan Liu/2023 1.46 [1.16; 1.75]
Omitting Shengqun Jiang/2022 1.47 [1.18; 1.77]
Omitting Yafang Wei/2022 1.39 [1.12; 1.67]
Omitting Hongyan Li/2022 1.47 [1.17; 1.77]
Omitting Bing Zhou/2021 1.48 [1.18; 1.77]
Omitting Shuai Fu/2021 1.46 [1.16; 1.76]
Omitting Li Yao/2019 1.47 [1.17; 1.76]
Omitting Rong Liu/2025 1.44 [1.15; 1.74]
Omitting Yao Hu/2025 1.48 [1.18; 1.77]
Omitting Jianping Gong/2023 1.41 [1.13; 1.69]
Omitting Wenjuan Wang/2020 1.43 [1.14; 1.72]
Omitting Shushu Zhang/2024 1.42 [1.13; 1.71]
Omitting Dongzhu Zhang/2019 1.45 [1.16; 1.75]
Omitting Jun Li/2023 1.41 [1.12; 1.69]
Random effects model 1.45 [1.16; 1.74]
-1.5 -1 -0.5 0 0.5 1 1.5
Favours LBL Favours Mind Mapping
B Case analysis
Study
Standardised Mean Difference
SMD
95%-CI
Omitting Jun Liu/2025 1.38 [0.97; 1.79]
Omitting Hongjuan Shi/2023 1.37 [0.95; 1.78]
Omitting Shuo Wu/2022 1.17 [0.89; 1.45]
Omitting Jinyu Zhang/2025 1.35 [0.94; 1.77]
Omitting Ying Zhou/2025 1.35 [0.94; 1.77]
Omitting Qian Liu/2025 1.31 [0.90; 1.72]
Omitting Lin Guo/2025 1.38 [0.98; 1.79]
Omitting Xiaoxue Zhang/2025 1.29 [0.89; 1.68]
Omitting Yanming Zhang/2024 1.35 [0.93; 1.77]
Omitting Jia Chen/2024 1.36 [0.95; 1.77]
Omitting Yanqian Deng/2024 1.36 [0.94; 1.77]
Omitting Lingtao Liu/2023 1.34 [0.93; 1.75]
Omitting Bing Zhou/2021 1.39 [0.99; 1.79]
Omitting Shuai Fu/2021 1.37 [0.96; 1.78]
Omitting Li Yao/2019 1.40 [1.01; 1.79]
Omitting Wenjuan Wang/2020 1.27 [0.89; 1.65]
Omitting Qingsong Zhang/2025 1.31 [0.90; 1.71]
Random effects model 1.34 [0.95; 1.72]
-1.5 -1 -0.5 0 0.5 1 1.5
Favours LBL Favours Mind Mapping
C Procedural skill
Study
Standardised Mean Difference
SMD
95%-CI
Omitting Yuan Cheng/2019 1.71 [1.25; 2.18]
Omitting Shanshan Cui/2024 1.73 [1.27; 2.19]
Omitting Jiajia Fan/2021 1.69 [1.22; 2.16]
Omitting Jun Liu/2025 1.66 [1.19; 2.13]
Omitting Li Qu/2023 1.73 [1.27; 2.19]
Omitting Hongjuan Shi/2023 1.71 [1.25; 2.18]
Omitting Xin Liao/2022 1.64 [1.18; 2.10]
Omitting Le Zhang/2021 1.70 [1.23; 2.17]
Omitting Yan Xu/2018 1.66 [1.19; 2.12]
Omitting Jing Zhao/2017 1.70 [1.23; 2.17]
Omitting Lin Guo/2025 1.70 [1.23; 2.17]
Omitting Xiaoxue Zhang/2025 1.67 [1.20; 2.14]
Omitting Yue Yu/2024 1.72 [1.26; 2.19]
Omitting Ning Du/2024 1.70 [1.23; 2.17]
Omitting Min Tang/2024 1.72 [1.25; 2.18]
Omitting Jin Zhang/2024 1.70 [1.23; 2.17]
Omitting Wenjie Xu/2024 1.55 [1.16; 1.95]
Omitting Jingmin Dong/2023 1.71 [1.24; 2.18]
Omitting Shengqun Jiang/2022 1.71 [1.24; 2.18]
Omitting Bing Zhou/2021 1.73 [1.27; 2.19]
Omitting Li Yao/2019 1.73 [1.27; 2.19]
Omitting Rong Liu/2025 1.68 [1.21; 2.15]
Omitting Yao Hu/2025 1.71 [1.24; 2.18]
Omitting Jianping Gong/2023 1.59 [1.16; 2.02]
Omitting Wenjuan Wang/2020 1.66 [1.19; 2.13]
Omitting Dongzhu Zhang/2019 1.70 [1.23; 2.16]
Omitting Jun Li/2023 1.60 [1.16; 2.04]
Omitting Yuanhui Sun/2025 1.59 [1.16; 2.02]
Random effects model 1.68 [1.23; 2.13]
-2 -1 0 1 2
Favours LBL Favours Mind Mapping
D Level of theoretical knowledge
Study
Standardised Mean Difference
SMD
95%-CI
Omitting Yunfeng Jiang/2024 1.10 [0.56; 1.64]
Omitting Hongjuan Shi/2023 1.17 [0.66; 1.69]
Omitting Xin Liao/2022 0.99 [0.53; 1.45]
Omitting Jing Zhao/2017 1.14 [0.60; 1.68]
Omitting Qian Liu/2025 1.06 [0.54; 1.59]
Omitting Min Tang/2024 1.15 [0.62; 1.68]
Omitting Zhouwei Xu/2025 1.13 [0.58; 1.68]
Omitting Wenjuan Wang/2020 1.20 [0.76; 1.64]
Omitting Yuehua Pu/2017 0.93 [0.57; 1.28]
Random effects model 1.09 [0.63; 1.55]
-1.5 -1 -0.5 0 0.5 1 1.5
Favours LBL Favours Mind Mapping
E Clinical reasoning
Study
Standardised Mean Difference
SMD
95%-CI
Omitting Le Zhang/2021 2.03 [0.88; 3.17]
Omitting Jinyu Zhang/2025 2.08 [0.95; 3.21]
Omitting Yue Yu/2024 2.04 [0.90; 3.19]
Omitting Min Tang/2024 2.07 [0.94; 3.20]
Omitting Jin Zhang/2024 2.00 [0.84; 3.15]
Omitting Yanqian Deng/2024 2.05 [0.91; 3.19]
Omitting Shengqun Jiang/2022 1.40 [0.96; 1.85]
Omitting Zhouwei Xu/2025 2.07 [0.94; 3.20]
Omitting Rong Liu/2025 2.01 [0.85; 3.16]
Omitting Yao Hu/2025 2.06 [0.92; 3.20]
Omitting Jianping Gong/2023 1.93 [0.78; 3.09]
Omitting Wenjuan Wang/2020 1.95 [0.80; 3.11]
Omitting Yuehua Pu/2017 1.77 [0.73; 2.81]
Random effects model 1.96 [0.91; 3.00]
-3 -2 -1 0 1 2 3
Favours LBL Favours Mind Mapping
F Learning motivation
Study
Standardised Mean Difference
SMD
95%-CI
Omitting Yi Guo/2020 1.87 [1.02; 2.71]
Omitting Min Lv/2025 1.82 [0.96; 2.67]
Omitting Xin Liao/2022 1.74 [0.91; 2.58]
Omitting Le Zhang/2021 1.86 [1.01; 2.71]
Omitting Jing Zhao/2017 1.82 [0.96; 2.68]
Omitting Ying Zhou/2025 1.89 [1.05; 2.72]
Omitting Qian Liu/2025 1.87 [1.02; 2.72]
Omitting Yue Yu/2024 1.84 [0.98; 2.70]
Omitting Min Tang/2024 1.88 [1.04; 2.72]
Omitting Jin Zhang/2024 1.86 [1.01; 2.71]
Omitting Yanqian Deng/2024 1.88 [1.03; 2.72]
Omitting Shengqun Jiang/2022 1.44 [1.11; 1.78]
Omitting Zhouwei Xu/2025 1.84 [0.98; 2.70]
Omitting Rong Liu/2025 1.85 [1.00; 2.70]
Omitting Wenjuan Wang/2020 1.73 [0.90; 2.56]
Random effects model 1.80 [1.02; 2.58]
-2 -1 0 1 2
Favours LBL Favours Mind Mapping
G Autonomous learning ability
Study
Standardised Mean Difference
SMD
95%-CI
Omitting Yi Guo/2020 1.77 [0.34; 3.21]
Omitting Jun Liu/2025 2.00 [0.60; 3.41]
Omitting Hongjuan Shi/2023 1.99 [0.58; 3.40]
Omitting Le Zhang/2021 1.88 [0.43; 3.33]
Omitting Jing Zhao/2017 1.96 [0.52; 3.39]
Omitting Xiaoxue Zhang/2025 1.86 [0.41; 3.32]
Omitting Jin Zhang/2024 1.89 [0.43; 3.34]
Omitting Yanqian Deng/2024 1.94 [0.50; 3.38]
Omitting Shengqun Jiang/2022 1.10 [0.75; 1.46]
Random effects model 1.82 [0.56; 3.09]
-3 -2 -1 0 1 2 3
Favours LBL Favours Mind Mapping
H Problem-solving ability
Study
Standardised Mean Difference
SMD
95%-CI
Omitting Yi Guo/2020 2.27 [1.01; 3.52]
Omitting Min Lv/2025 2.43 [1.17; 3.69]
Omitting Hongjuan Shi/2023 2.48 [1.24; 3.71]
Omitting Xin Liao/2022 2.38 [1.11; 3.66]
Omitting Qian Liu/2025 2.45 [1.21; 3.70]
Omitting Yue Yu/2024 2.44 [1.19; 3.70]
Omitting Jin Zhang/2024 2.40 [1.13; 3.67]
Omitting Shengqun Jiang/2022 1.75 [1.24; 2.26]
Omitting Rong Liu/2025 2.45 [1.21; 3.70]
Omitting Qingsong Zhang/2025 2.39 [1.12; 3.66]
Omitting Yuanhui Sun/2025 2.17 [0.97; 3.38]
Random effects model 2.32 [1.19; 3.46]
-3 -2 -1 0 1 2 3
Favours LBL Favours Mind Mapping
I Proficiency in literature retrieval
Study
Standardised Mean Difference
SMD
95%-CI
Omitting Yi Guo/2020 0.99 [0.67; 1.31]
Omitting Hongjuan Shi/2023 1.48 [0.63; 2.34]
Omitting Qian Liu/2025 1.43 [0.45; 2.41]
Omitting Jin Zhang/2024 1.33 [0.29; 2.38]
Random effects model 1.24 [0.63; 1.86]
-2 -1 0 1 2
Favours LBL Favours Mind Mapping
J Clinical skills
Study
Standardised Mean Difference
SMD
95%-CI
Omitting Yunfeng Jiang/2024 1.55 [0.58; 2.51]
Omitting Le Zhang/2021 1.57 [0.62; 2.51]
Omitting Yue Yu/2024 1.41 [0.46; 2.36]
Omitting Rong Liu/2025 1.43 [0.47; 2.38]
Omitting Yao Hu/2025 1.77 [1.12; 2.42]
Omitting Qingsong Zhang/2025 1.47 [0.51; 2.44]
Omitting Yuanhui Sun/2025 1.17 [0.51; 1.84]
Random effects model 1.48 [0.66; 2.29]
-2 -1 0 1 2
Favours LBL Favours Mind Mapping
K Teamwork
Study
Standardised Mean Difference
SMD
95%-CI
Omitting Hongjuan Shi/2023 2.39 [0.18; 4.59]
Omitting Le Zhang/2021 2.30 [0.06; 4.54]
Omitting Qian Liu/2025 2.28 [0.02; 4.53]
Omitting Min Tang/2024 2.44 [0.27; 4.61]
Omitting Shengqun Jiang/2022 1.25 [0.73; 1.76]
Omitting Zhouwei Xu/2025 2.17 [-0.10; 4.44]
Omitting Yao Hu/2025 2.38 [0.17; 4.59]
Random effects model 2.15 [0.27; 4.03]
-4 -2 0 2 4
Favours LBL Favours Mind Mapping
L course satisfaction
Study
Standardised Mean Difference
SMD
95%-CI
Omitting Yi Guo/2020 2.04 [0.89; 3.20]
Omitting Le Zhang/2021 2.00 [0.83; 3.17]
Omitting Ying Zhou/2025 2.06 [0.91; 3.21]
Omitting Xiaoxue Zhang/2025 1.94 [0.76; 3.11]
Omitting Yue Yu/2024 2.01 [0.84; 3.18]
Omitting Ning Du/2024 2.03 [0.87; 3.20]
Omitting Min Tang/2024 2.05 [0.90; 3.21]
Omitting Shengqun Jiang/2022 1.42 [0.90; 1.94]
Omitting Zhouwei Xu/2025 2.00 [0.82; 3.17]
Omitting Wenjuan Wang/2020 2.07 [0.92; 3.21]
Omitting Yuehua Pu/2017 1.83 [0.69; 2.97]
Omitting Jun Li/2023 1.82 [0.68; 2.95]
Random effects model 1.93 [0.87; 3.00]
-3 -2 -1 0 1 2 3
Favours LBL Favours Mind Mapping
