# Supplementary material for: The effectiveness of mind mapping versus lecture-based learning in medical education of China’s standardized residency training: a systematic review and meta-analysis of randomized controlled studies
Source: Front Med (Lausanne). 2026 May 5;13:1789650. doi: 10.3389/fmed.2026.1789650 (PMC13183817; doi:10.3389/fmed.2026.1789650)
Supplement: Supplementary Figure S2 — Subgroup meta-analyses of different departments and teaching methods on examination scores results. The meta-analyses on the effect of mind mapping with the subgroup analyses of different departments on (A) theoretical knowledge scores, (B) case analysis scores, (C) procedural skill scores; the meta-analyses on the effect of mind mapping with the subgroup analyses of only mind mapping group and mind mapping combined with other teaching methods on (D) theoretical knowledge scores, (E) case analysis scores and (F) procedural skill scores; the meta-analyses on effect of mind mapping with the subgroup analyses of different methods of generating mind map on the (G) theoretical knowledge scores, (H) case analysis scores and (I) procedural skill scores; the meta-analyses on the effect of mind mapping with the subgroup analyses of different intervention duration on (J) theoretical knowledge scores, (K) case analysis scores and (L) procedural skill scores. The large diamond at the bottle of the plot represents the pooled SMD of all studies. The width of the diamond represents with 95%CI. [file Image_2.pdf]

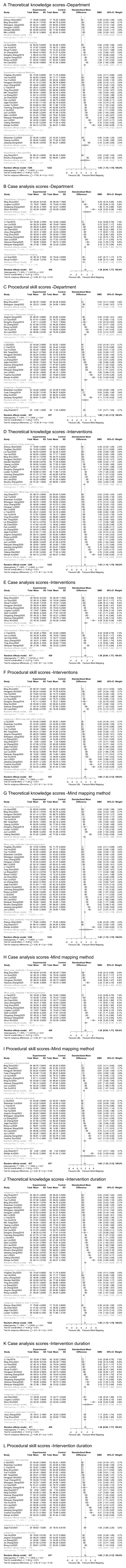
A Theoretical knowledge scores -Department
B Case analysis scores -Department
C Procedural skill scores -Department
D Theoretical knowledge scores -Interventions
E Case analysis scores -Interventions
F Procedural skill scores -Interventions
G Theoretical knowledge scores -Mind mapping method
H Case analysis scores -Mind mapping method
I Procedural skill scores -Mind mapping method
J Theoretical knowledge scores -Intervention duration
K Case analysis scores -Intervention duration
L Procedural skill scores -Intervention duration
