# Supplementary material for: The effectiveness of mind mapping versus lecture-based learning in medical education of China’s standardized residency training: a systematic review and meta-analysis of randomized controlled studies
Source: Front Med (Lausanne). 2026 May 5;13:1789650. doi: 10.3389/fmed.2026.1789650 (PMC13183817; doi:10.3389/fmed.2026.1789650)
Supplement: Supplementary Figure S3 — Forest plots of continuous endpoints of questionnaire surveys results. The meta-analyses on the effect of mind mapping on the level of (A) theoretical knowledge, (B) clinical reasoning, (C) learning motivation, (D) autonomous learning ability, (E) problem-solving ability, (F) proficiency in literature retrieval, (G) clinical skills, (H) teamwork, and (I) course satisfaction. The large diamond at the bottle of the plot represents the pooled SMD of all studies. The width of the diamond represents with 95%CI. The black horizontal bar represents the 95% prediction interval. [file Image_3.pdf]

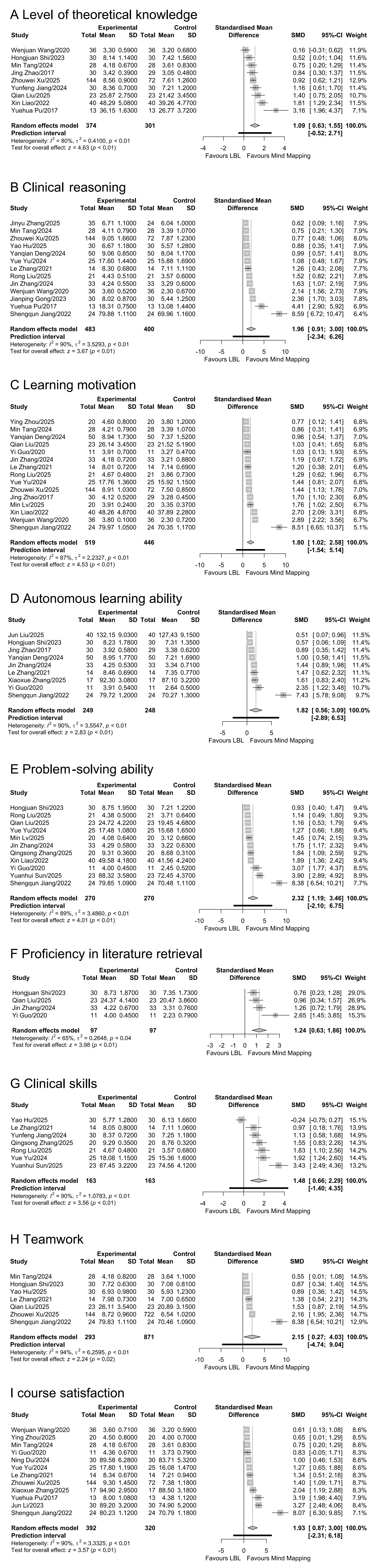
A Level of theoretical knowledge
Study | Experimental Total Mean SD | Control Total Mean SD | Standardised Mean Difference | SMD | 95%-CI | Weight
Wenjuan Wang/2020 36 3.30 0.5900 36 3.20 0.6800 0.16 [-0.31; 0.62] 11.9%
Hongjuan Shi/2023 30 8.14 1.1400 30 7.42 1.5600 0.52 [0.01; 1.04] 11.6%
Min Tang/2024 28 4.18 0.6700 28 3.61 0.8300 0.75 [0.20; 1.29] 11.4%
Jing Zhao/2017 30 3.42 0.3900 29 3.05 0.4800 0.84 [0.30; 1.37] 11.5%
Zhouwei Xu/2025 144 8.56 0.9000 72 7.61 1.2600 0.92 [0.62; 1.21] 12.9%
Yunfeng Jiang/2024 30 8.36 0.7000 30 7.21 1.2000 1.16 [0.61; 1.70] 11.4%
Qian Liu/2025 23 25.87 2.7500 23 21.42 3.4500 1.40 [0.75; 2.05] 10.7%
Xin Liao/2022 40 48.29 5.0800 40 39.26 4.7700 1.81 [1.29; 2.34] 11.5%
Yuehua Pu/2017 13 36.15 1.6300 13 26.77 3.7200 3.16 [1.96; 4.37] 7.1%
Random effects model 374 301 1.09 [0.63; 1.55] 100.0%
Prediction interval [-0.52; 2.71]
Heterogeneity: I² = 80%, τ² = 0.4100, p < 0.01
Test for overall effect: z = 4.63 (p < 0.01)
-4 -2 0 2 4
Favours LBL Favours Mind Mapping
B Clinical reasoning
Study | Experimental Total Mean SD | Control Total Mean SD | Standardised Mean Difference | SMD | 95%-CI | Weight
Jinyu Zhang/2025 35 6.71 1.1000 24 6.04 1.0000 0.62 [0.09; 1.16] 7.9%
Min Tang/2024 28 4.11 0.7900 28 3.39 1.0700 0.75 [0.21; 1.30] 7.9%
Zhouwei Xu/2025 144 9.05 1.6600 72 7.87 1.2300 0.77 [0.48; 1.06] 8.0%
Yao Hu/2025 30 6.67 1.1800 30 5.57 1.2800 0.88 [0.35; 1.41] 7.9%
Yanqian Deng/2024 50 9.06 0.8500 50 8.04 1.1700 0.99 [0.57; 1.41] 8.0%
Yue Yu/2024 25 17.60 1.4400 25 15.88 1.6900 1.08 [0.48; 1.67] 7.9%
Le Zhang/2021 14 8.30 0.6800 14 7.11 1.1100 1.26 [0.43; 2.08] 7.7%
Rong Liu/2025 21 4.43 0.5100 21 3.57 0.6000 1.52 [0.82; 2.21] 7.8%
Jin Zhang/2024 33 4.24 0.5500 33 3.29 0.6000 1.63 [1.07; 2.19] 7.9%
Wenjuan Wang/2020 36 3.60 0.5200 36 2.30 0.6700 2.14 [1.56; 2.73] 7.9%
Jianping Gong/2023 30 8.02 0.8700 30 5.44 1.2500 2.36 [1.70; 3.03] 7.8%
Yuehua Pu/2017 13 18.31 0.7500 13 13.08 1.4400 4.41 [2.90; 5.92] 6.9%
Shengqun Jiang/2022 24 79.88 1.1100 24 69.96 1.1600 8.59 [6.72; 10.47] 6.4%
Random effects model 483 400 1.96 [0.91; 3.00] 100.0%
Prediction interval [-2.34; 6.26]
Heterogeneity: I² = 90%, τ² = 3.5293, p < 0.01
Test for overall effect: z = 3.67 (p < 0.01)
-10 -5 0 5 10
Favours LBL Favours Mind Mapping
C Learning motivation
Study | Experimental Total Mean SD | Control Total Mean SD | Standardised Mean Difference | SMD | 95%-CI | Weight
Ying Zhou/2025 20 4.60 0.8000 20 3.80 1.2000 0.77 [0.12; 1.41] 6.8%
Min Tang/2024 28 4.21 0.7900 28 3.39 1.0700 0.86 [0.31; 1.41] 6.9%
Yanqian Deng/2024 50 8.94 1.7300 50 7.37 1.5200 0.96 [0.54; 1.37] 7.0%
Qian Liu/2025 23 26.14 3.4500 23 21.52 5.1900 1.03 [0.41; 1.65] 6.8%
Yi Guo/2020 11 3.91 0.7000 11 3.27 0.4700 1.03 [0.13; 1.93] 6.5%
Jin Zhang/2024 33 4.18 0.7200 33 3.21 0.8800 1.19 [0.67; 1.72] 6.9%
Le Zhang/2021 14 8.01 0.7200 14 7.14 0.6900 1.20 [0.38; 2.01] 6.6%
Rong Liu/2025 21 4.67 0.4800 21 3.86 0.7300 1.29 [0.62; 1.96] 6.7%
Yue Yu/2024 25 17.76 1.3600 25 15.92 1.1500 1.44 [0.81; 2.07] 6.8%
Zhouwei Xu/2025 144 8.91 1.0300 72 7.50 0.8500 1.44 [1.13; 1.76] 7.0%
Jing Zhao/2017 30 4.12 0.5200 29 3.28 0.4500 1.70 [1.10; 2.30] 6.8%
Min Lv/2025 20 3.91 0.2400 20 3.35 0.3700 1.76 [1.02; 2.50] 6.7%
Xin Liao/2022 40 48.26 4.8700 40 37.89 2.2800 2.70 [2.09; 3.31] 6.8%
Wenjuan Wang/2020 36 3.80 0.1000 36 2.30 0.7200 2.89 [2.22; 3.56] 6.7%
Shengqun Jiang/2022 24 79.97 1.0500 24 70.35 1.1700 8.51 [6.65; 10.37] 5.1%
Random effects model 519 446 1.80 [1.02; 2.58] 100.0%
Prediction interval [-1.54; 5.14]
Heterogeneity: I² = 87%, τ² = 2.2327, p < 0.01
Test for overall effect: z = 4.53 (p < 0.01)
-10 -5 0 5 10
Favours LBL Favours Mind Mapping
D Autonomous learning ability
Study | Experimental Total Mean SD | Control Total Mean SD | Standardised Mean Difference | SMD | 95%-CI | Weight
Jun Liu/2025 40 132.15 9.0300 40 127.43 9.1500 0.51 [0.07; 0.96] 11.5%
Hongjuan Shi/2023 30 8.23 1.7800 30 7.31 1.3500 0.57 [0.06; 1.09] 11.4%
Jing Zhao/2017 30 3.92 0.5800 29 3.38 0.6200 0.89 [0.35; 1.42] 11.4%
Yanqian Deng/2024 50 8.95 1.7700 50 7.21 1.6900 1.00 [0.58; 1.41] 11.5%
Jin Zhang/2024 33 4.25 0.5300 33 3.34 0.7100 1.44 [0.89; 1.98] 11.4%
Le Zhang/2021 14 8.46 0.6900 14 7.35 0.7700 1.47 [0.62; 2.32] 11.1%
Xiaoxue Zhang/2025 17 92.30 3.0800 17 87.10 3.2200 1.61 [0.83; 2.40] 11.2%
Yi Guo/2020 11 4.00 0.4500 11 2.64 0.5000 2.35 [1.22; 3.48] 10.7%
Shengqun Jiang/2022 24 79.72 1.2000 24 70.27 1.3000 7.43 [5.78; 9.08] 9.7%
Random effects model 249 248 1.82 [0.56; 3.09] 100.0%
Prediction interval [-2.89; 6.53]
Heterogeneity: I² = 90%, τ² = 3.5547, p < 0.01
Test for overall effect: z = 2.83 (p < 0.01)
-5 0 5
Favours LBL Favours Mind Mapping
E Problem-solving ability
Study | Experimental Total Mean SD | Control Total Mean SD | Standardised Mean Difference | SMD | 95%-CI | Weight
Hongjuan Shi/2023 30 8.75 1.9500 30 7.21 1.2200 0.93 [0.40; 1.47] 9.4%
Rong Liu/2025 21 4.38 0.5000 21 3.71 0.6400 1.14 [0.49; 1.80] 9.3%
Qian Liu/2025 23 24.72 4.2200 23 19.45 4.6800 1.16 [0.53; 1.79] 9.4%
Yue Yu/2024 25 17.48 1.0800 25 15.68 1.6500 1.27 [0.66; 1.88] 9.4%
Min Lv/2025 20 4.08 0.6400 20 3.12 0.6600 1.45 [0.74; 2.15] 9.3%
Jin Zhang/2024 33 4.29 0.5800 33 3.22 0.6300 1.75 [1.17; 2.32] 9.4%
Qingsong Zhang/2025 20 9.31 0.3600 20 8.68 0.3100 1.84 [1.09; 2.59] 9.2%
Xin Liao/2022 40 49.58 4.1800 40 41.56 4.2400 1.89 [1.36; 2.42] 9.4%
Yi Guo/2020 11 4.00 0.4500 11 2.45 0.5200 3.07 [1.77; 4.37] 8.5%
Yuanhui Sun/2025 23 88.32 3.5800 23 72.45 4.3700 3.90 [2.89; 4.92] 8.9%
Shengqun Jiang/2022 24 79.85 1.0900 24 70.48 1.1100 8.38 [6.54; 10.21] 7.7%
Random effects model 270 270 2.32 [1.19; 3.46] 100.0%
Prediction interval [-2.10; 6.75]
Heterogeneity: I² = 89%, τ² = 3.4860, p < 0.01
Test for overall effect: z = 4.01 (p < 0.01)
-10 -5 0 5 10
Favours LBL Favours Mind Mapping
F Proficiency in literature retrieval
Study | Experimental Total Mean SD | Control Total Mean SD | Standardised Mean Difference | SMD | 95%-CI | Weight
Hongjuan Shi/2023 30 8.73 1.8700 30 7.35 1.7300 0.76 [0.23; 1.28] 29.0%
Qian Liu/2025 23 24.37 4.1400 23 20.47 3.8600 0.96 [0.34; 1.57] 26.9%
Jin Zhang/2024 33 4.22 0.6700 33 3.31 0.7600 1.26 [0.72; 1.79] 28.9%
Yi Guo/2020 11 4.00 0.4500 11 2.23 0.7900 2.65 [1.45; 3.85] 15.3%
Random effects model 97 97 1.24 [0.63; 1.86] 100.0%
Heterogeneity: I² = 65%, τ² = 0.2648, p = 0.04
Test for overall effect: z = 3.98 (p < 0.01)
-3 -2 -1 0 1 2 3
Favours LBL Favours Mind Mapping
G Clinical skills
Study | Experimental Total Mean SD | Control Total Mean SD | Standardised Mean Difference | SMD | 95%-CI | Weight
Yao Hu/2025 30 5.77 1.2800 30 6.13 1.6600 -0.24 [-0.75; 0.27] 15.1%
Le Zhang/2021 14 8.05 0.8000 14 7.11 1.0600 0.97 [0.18; 1.76] 13.9%
Yunfeng Jiang/2024 30 8.37 0.7200 30 7.25 1.1800 1.13 [0.58; 1.68] 14.9%
Qingsong Zhang/2025 20 9.29 0.3500 20 8.76 0.3200 1.55 [0.83; 2.26] 14.3%
Rong Liu/2025 21 4.67 0.4800 21 3.57 0.6800 1.83 [1.10; 2.56] 14.2%
Yue Yu/2024 25 18.08 1.1500 25 15.36 1.6000 1.92 [1.24; 2.60] 14.4%
Yuanhui Sun/2025 23 87.45 3.2200 23 74.56 4.1200 3.43 [2.49; 4.36] 13.2%
Random effects model 163 163 1.48 [0.66; 2.29] 100.0%
Prediction interval [-1.40; 4.35]
Heterogeneity: I² = 90%, τ² = 1.0783, p < 0.01
Test for overall effect: z = 3.56 (p < 0.01)
-4 -2 0 2 4
Favours LBL Favours Mind Mapping
H Teamwork
Study | Experimental Total Mean SD | Control Total Mean SD | Standardised Mean Difference | SMD | 95%-CI | Weight
Min Tang/2024 28 4.18 0.8200 28 3.64 1.1000 0.55 [0.01; 1.08] 14.5%
Hongjuan Shi/2023 30 7.72 0.6300 30 7.08 0.8100 0.87 [0.34; 1.40] 14.5%
Yao Hu/2025 30 6.93 0.9800 30 5.93 1.2300 0.89 [0.36; 1.42] 14.5%
Le Zhang/2021 14 7.98 0.7300 14 7.00 0.6500 1.38 [0.54; 2.21] 14.3%
Qian Liu/2025 23 26.11 3.5400 23 20.89 3.1500 1.53 [0.87; 2.19] 14.5%
Zhouwei Xu/2025 144 8.72 0.9600 722 6.54 1.0200 2.16 [1.95; 2.36] 14.7%
Shengqun Jiang/2022 24 79.83 1.1100 24 70.46 1.0900 8.38 [6.54; 10.21] 12.9%
Random effects model 293 871 2.15 [0.27; 4.03] 100.0%
Prediction interval [-4.74; 9.04]
Heterogeneity: I² = 94%, τ² = 6.2595, p < 0.01
Test for overall effect: z = 2.24 (p = 0.02)
-10 -5 0 5 10
Favours LBL Favours Mind Mapping
I course satisfaction
Study | Experimental Total Mean SD | Control Total Mean SD | Standardised Mean Difference | SMD | 95%-CI | Weight
Wenjuan Wang/2020 36 3.60 0.7100 36 3.20 0.5900 0.61 [0.13; 1.08] 8.6%
Ying Zhou/2025 20 4.50 0.8000 20 4.00 0.7000 0.65 [0.01; 1.29] 8.5%
Min Tang/2024 28 4.18 0.6700 28 3.61 0.8300 0.75 [0.20; 1.29] 8.6%
Yi Guo/2020 11 4.36 0.6700 11 3.73 0.7900 0.83 [-0.05; 1.71] 8.3%
Ning Du/2024 30 89.58 6.2800 30 83.71 5.3200 1.00 [0.46; 1.53] 8.6%
Yue Yu/2024 25 17.80 1.1900 25 16.08 1.4700 1.27 [0.65; 1.88] 8.6%
Le Zhang/2021 14 8.34 0.6700 14 7.21 0.9400 1.34 [0.51; 2.18] 8.3%
Zhouwei Xu/2025 144 9.30 1.4500 72 7.38 1.1800 1.40 [1.09; 1.71] 8.7%
Xiaoxue Zhang/2025 17 94.90 2.9500 17 88.50 3.1800 2.04 [1.19; 2.88] 8.3%
Yuehua Pu/2017 13 8.00 1.0800 13 4.38 1.1200 3.19 [1.98; 4.40] 7.9%
Jun Li/2023 30 89.20 3.2000 30 74.90 5.2000 3.27 [2.48; 4.06] 8.4%
Shengqun Jiang/2022 24 80.23 1.1200 24 70.79 1.1800 8.07 [6.30; 9.85] 7.1%
Random effects model 392 320 1.93 [0.87; 3.00] 100.0%
Prediction interval [-2.31; 6.18]
Heterogeneity: I² = 90%, τ² = 3.3325, p < 0.01
Test for overall effect: z = 3.57 (p < 0.01)
-5 0 5
Favours LBL Favours Mind Mapping
