# Supplementary material for: The effectiveness of mind mapping versus lecture-based learning in medical education of China’s standardized residency training: a systematic review and meta-analysis of randomized controlled studies
Source: Front Med (Lausanne). 2026 May 5;13:1789650. doi: 10.3389/fmed.2026.1789650 (PMC13183817; doi:10.3389/fmed.2026.1789650)

A Clinical reasoning -Department

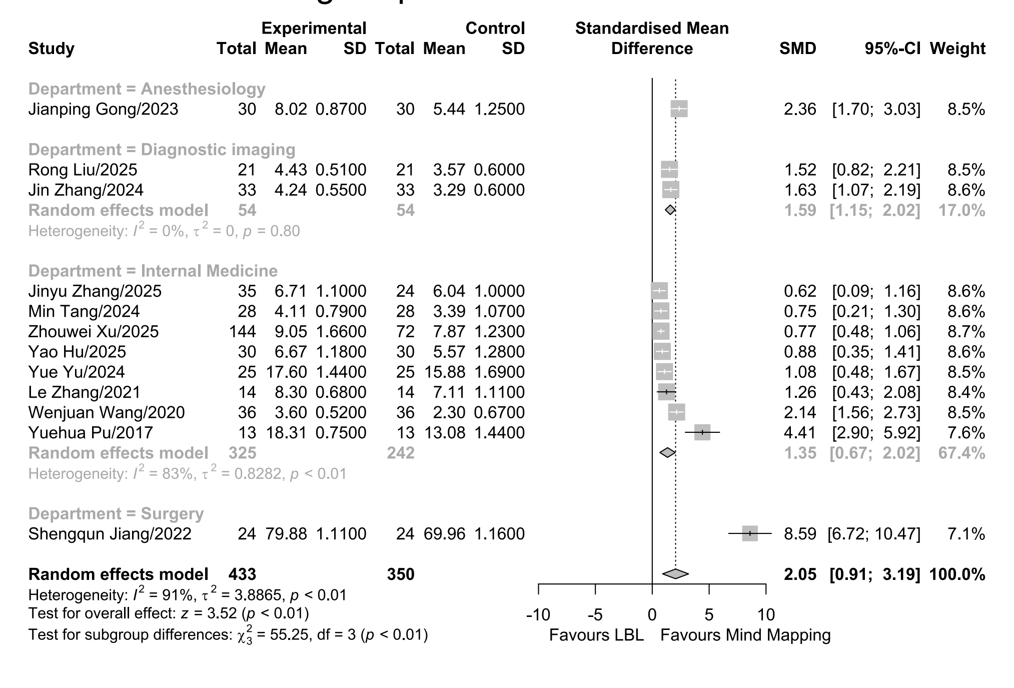

B Learning motivation -Department

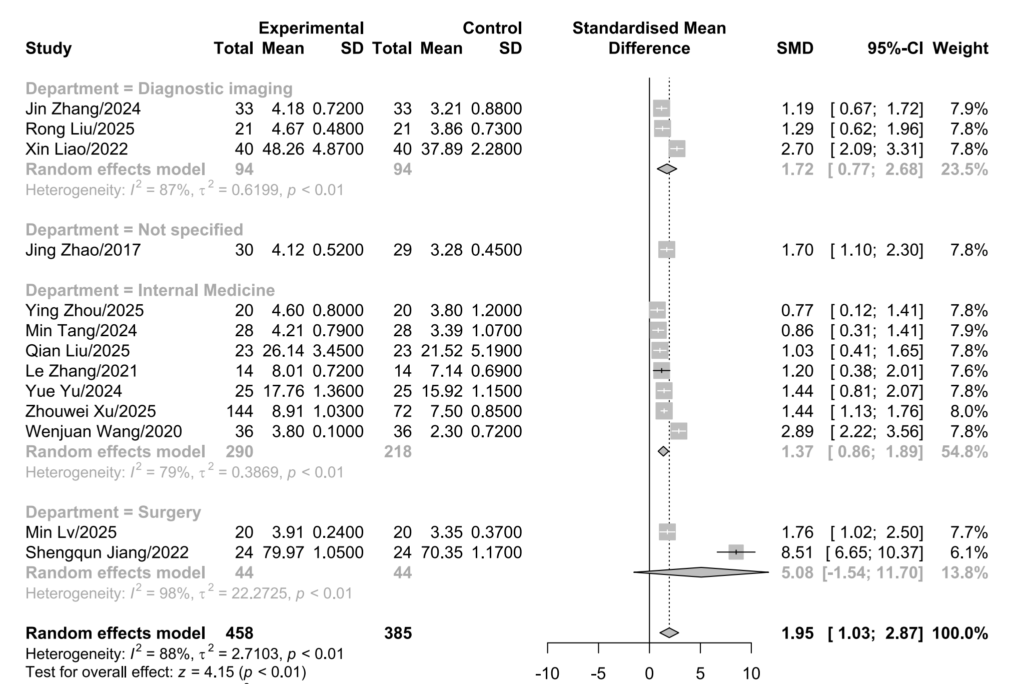

C Problem-solving ability -Department

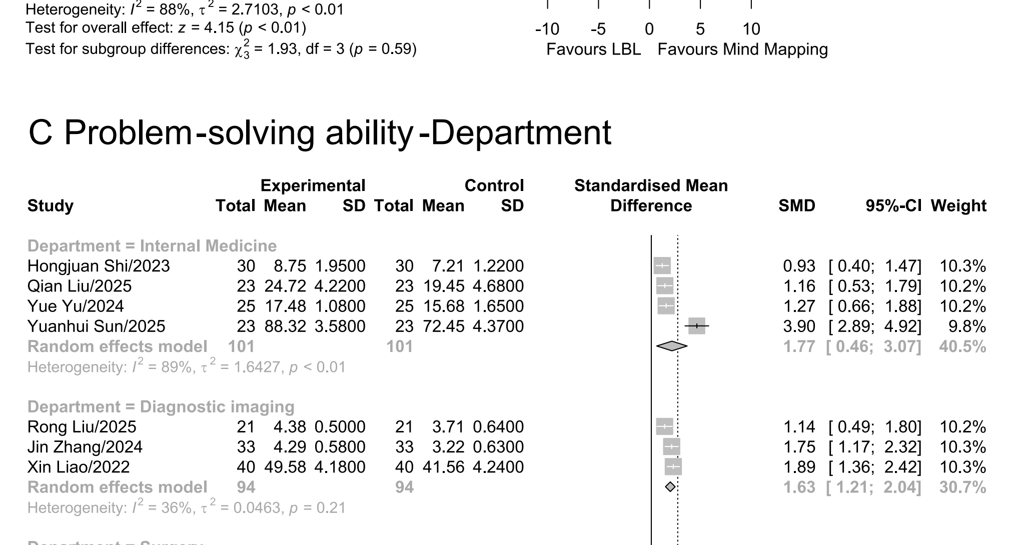

D Course satisfaction -Department

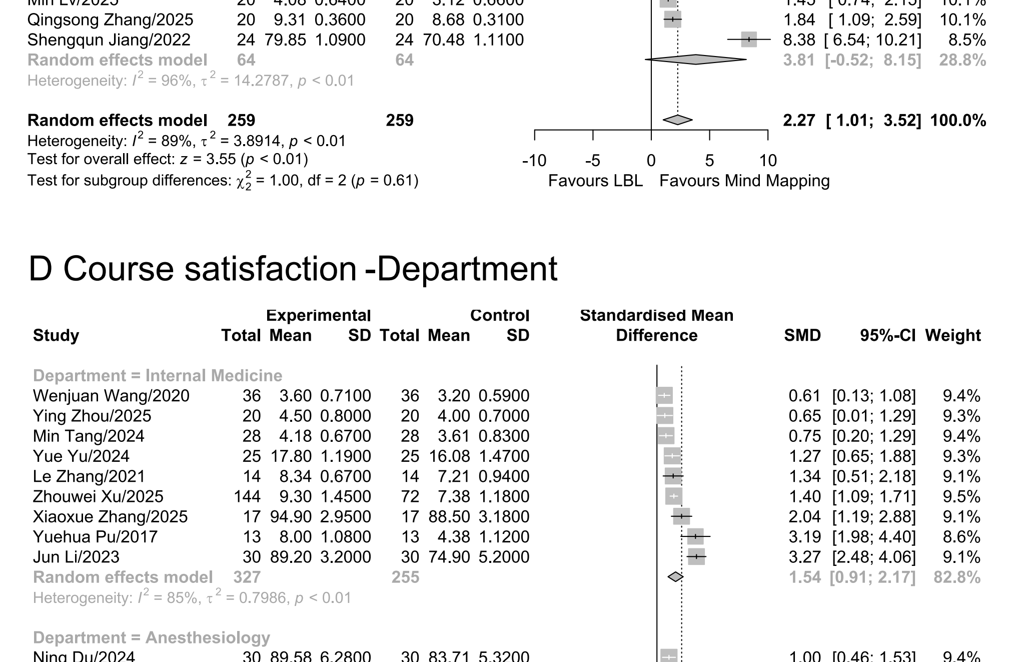

E Clinical reasoning -Interventions

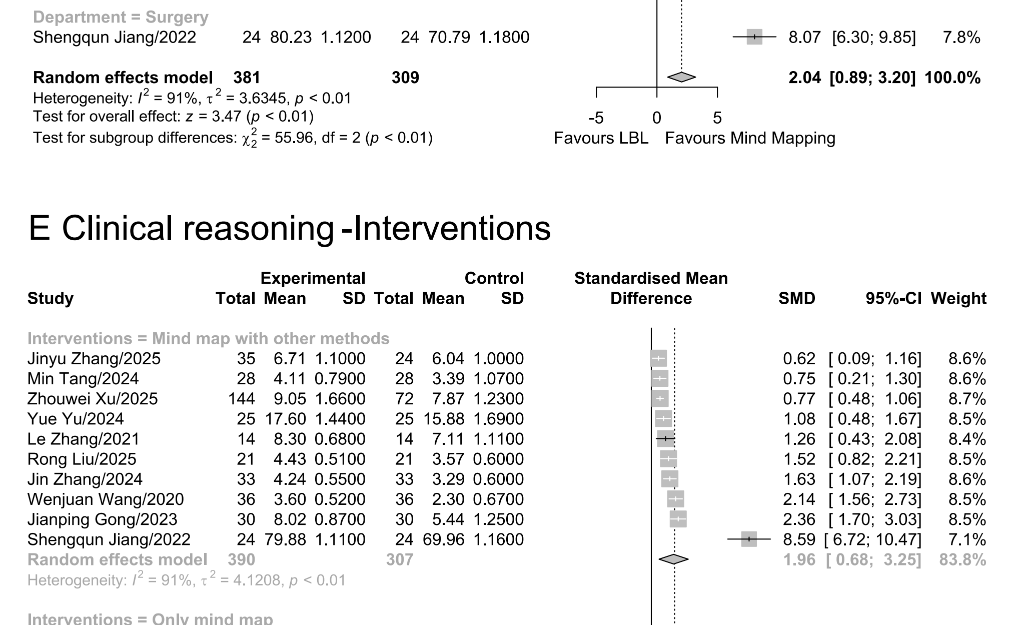

F Learning motivation -Interventions

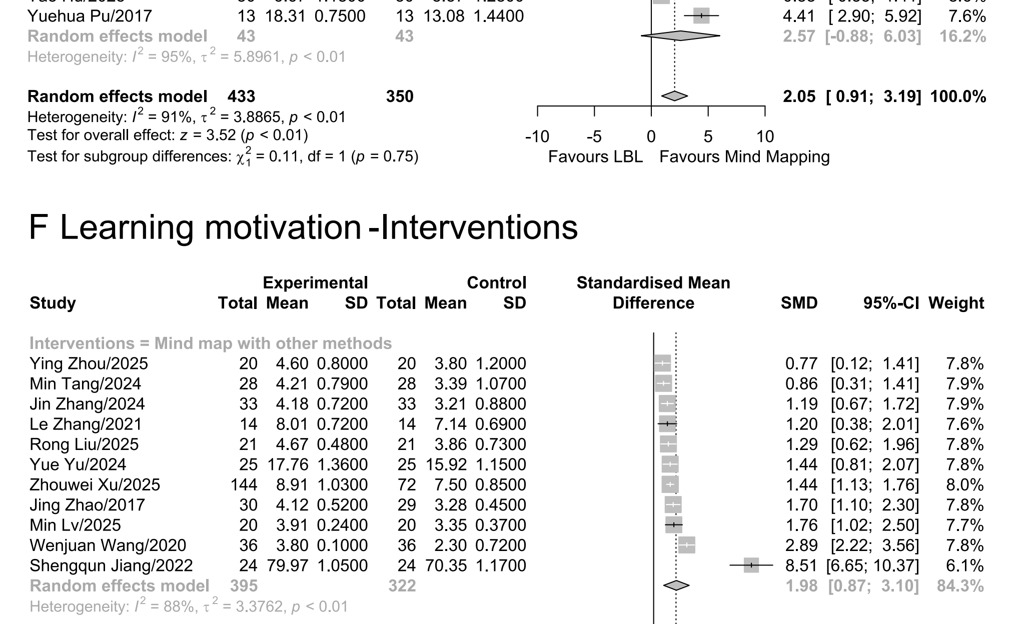

G Problem-solving ability -Interventions

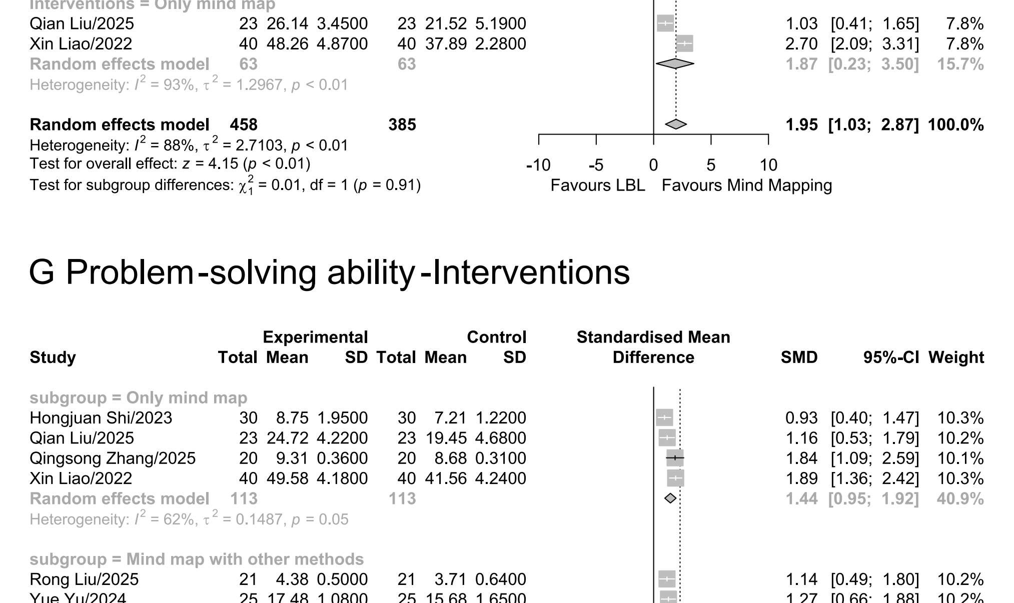

H Course satisfaction -Interventions

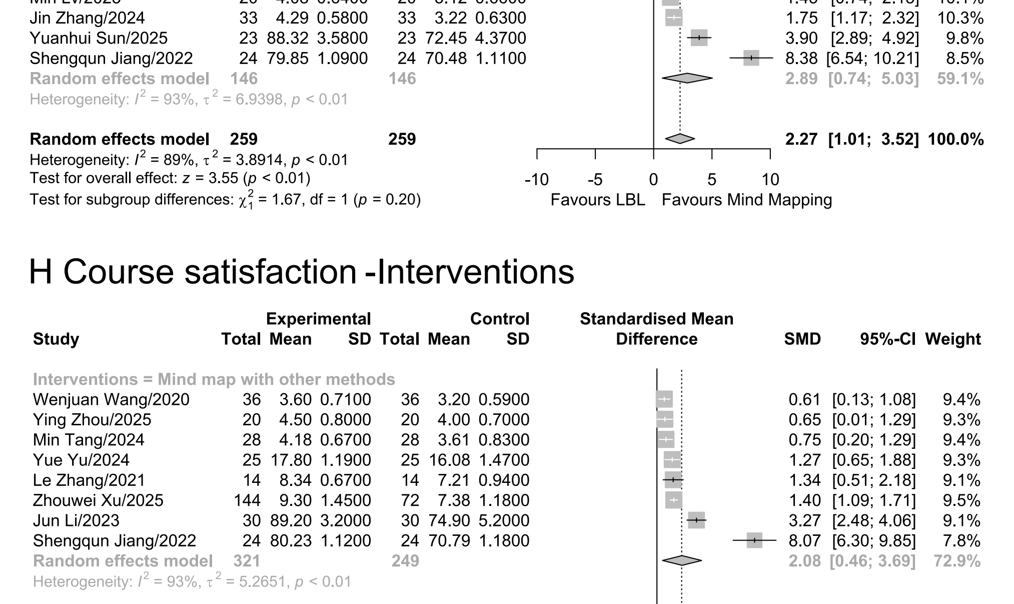

I Clinical reasoning -Mind mapping method

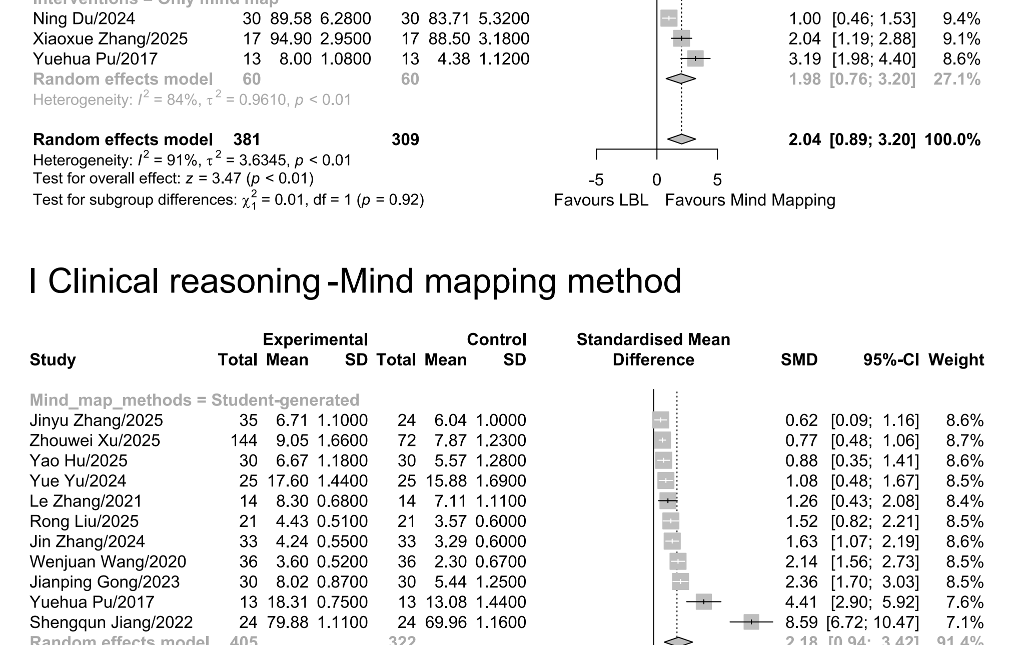

J Learning motivation -Mind mapping method

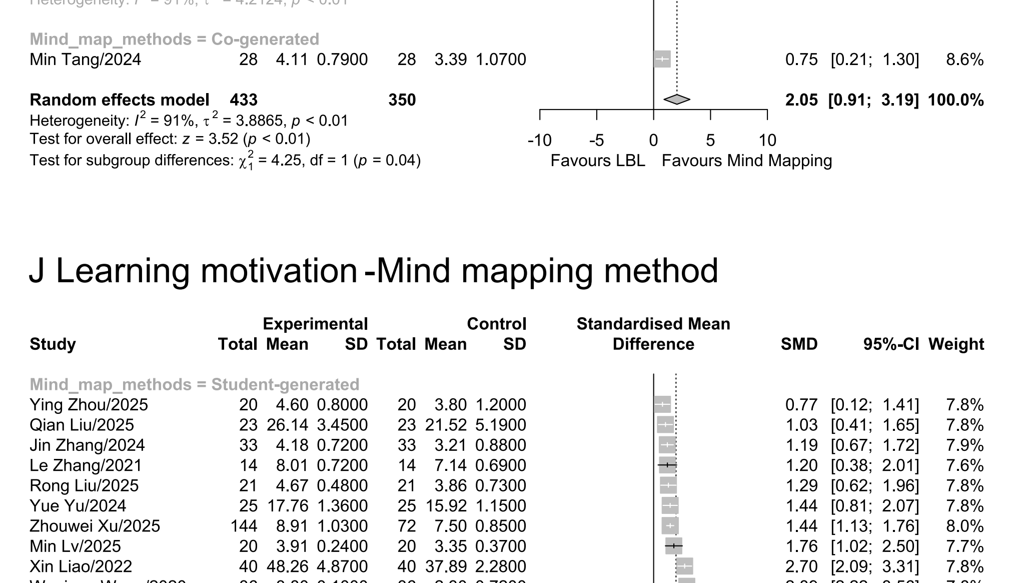

K Problem-solving ability -Mind mapping method

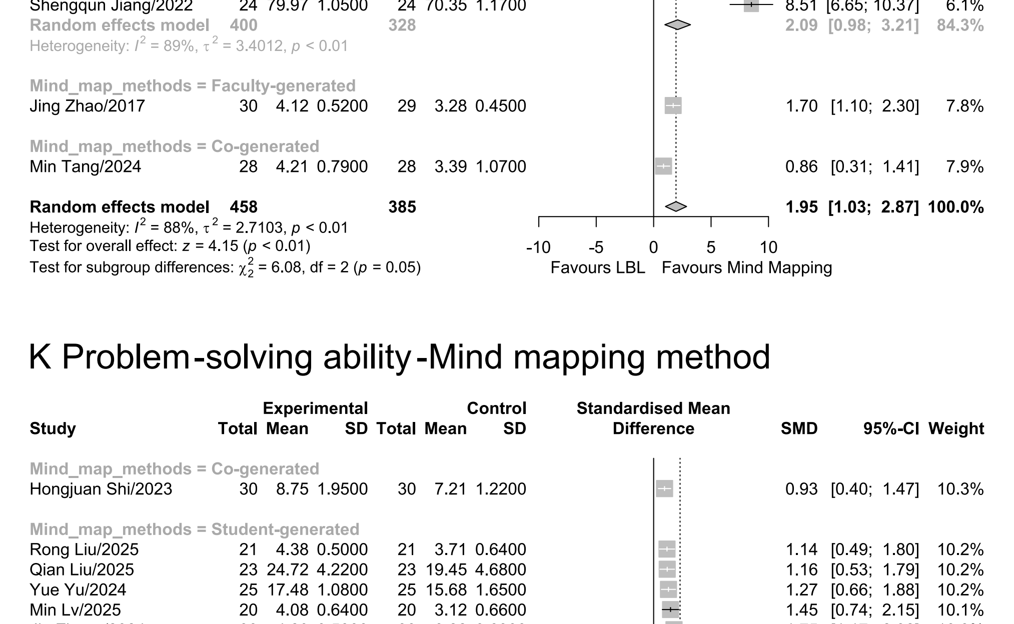

L Course satisfaction -Mind mapping method

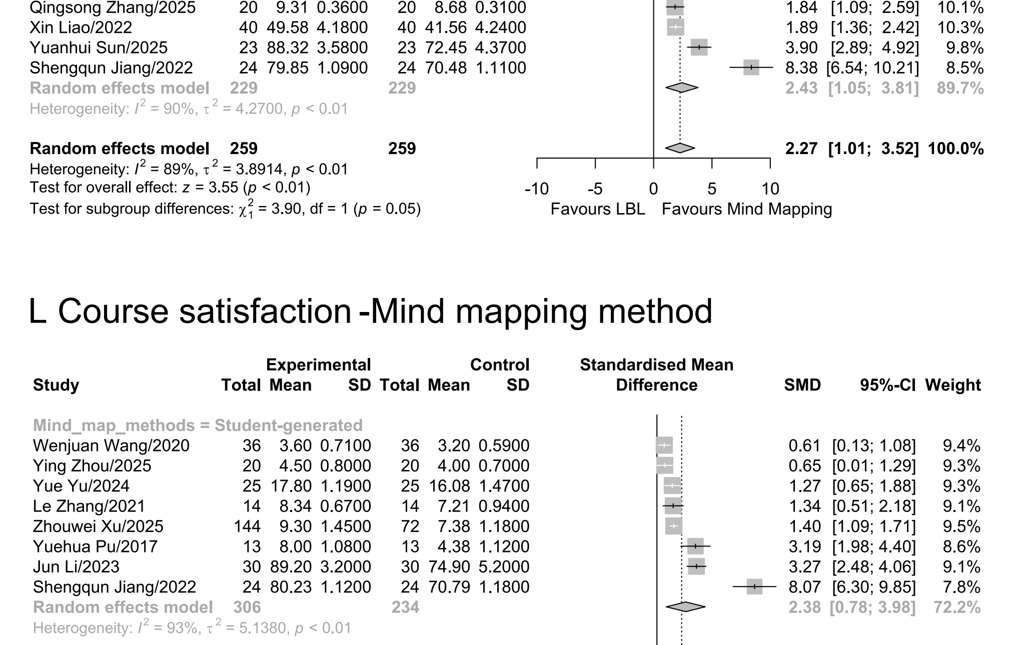

M Clinical reasoning -Intervention duration

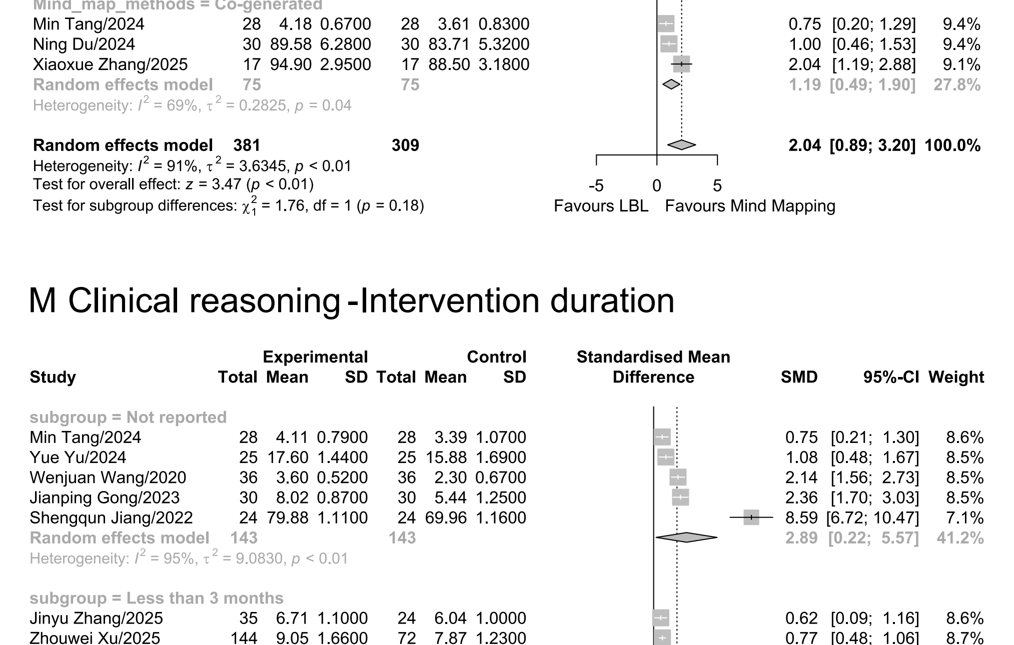

N Learning motivation -Intervention duration

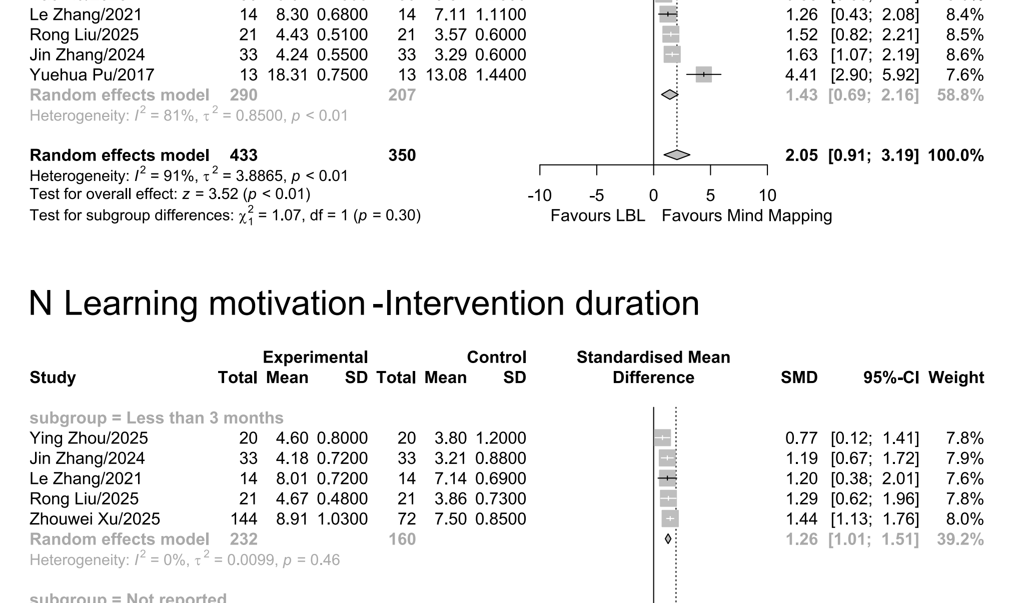

O Problem-solving ability -Intervention duration

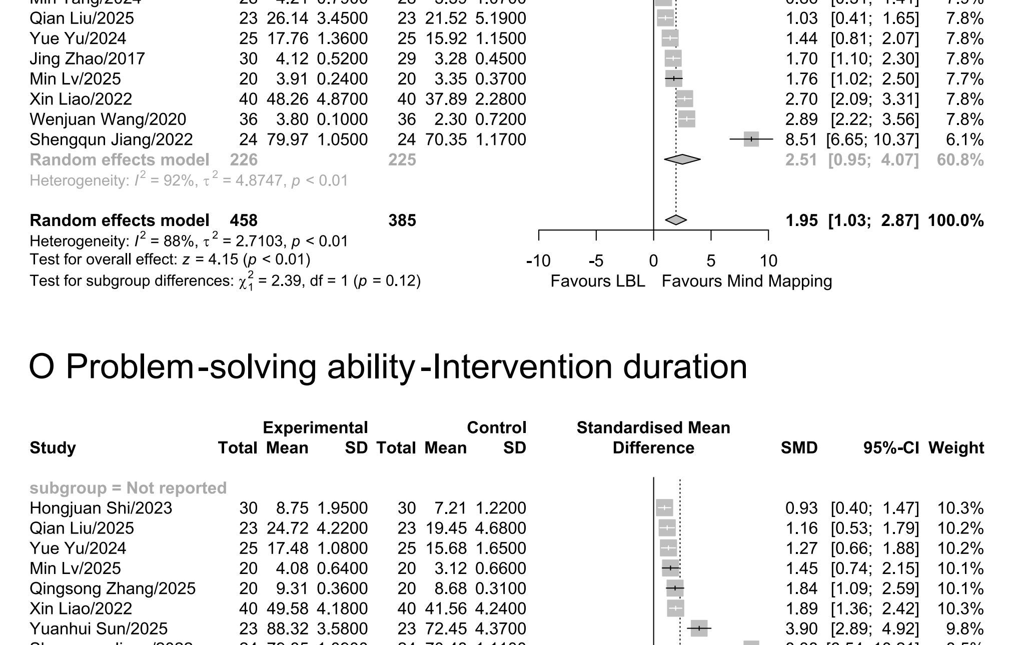

P Course satisfaction -Intervention duration

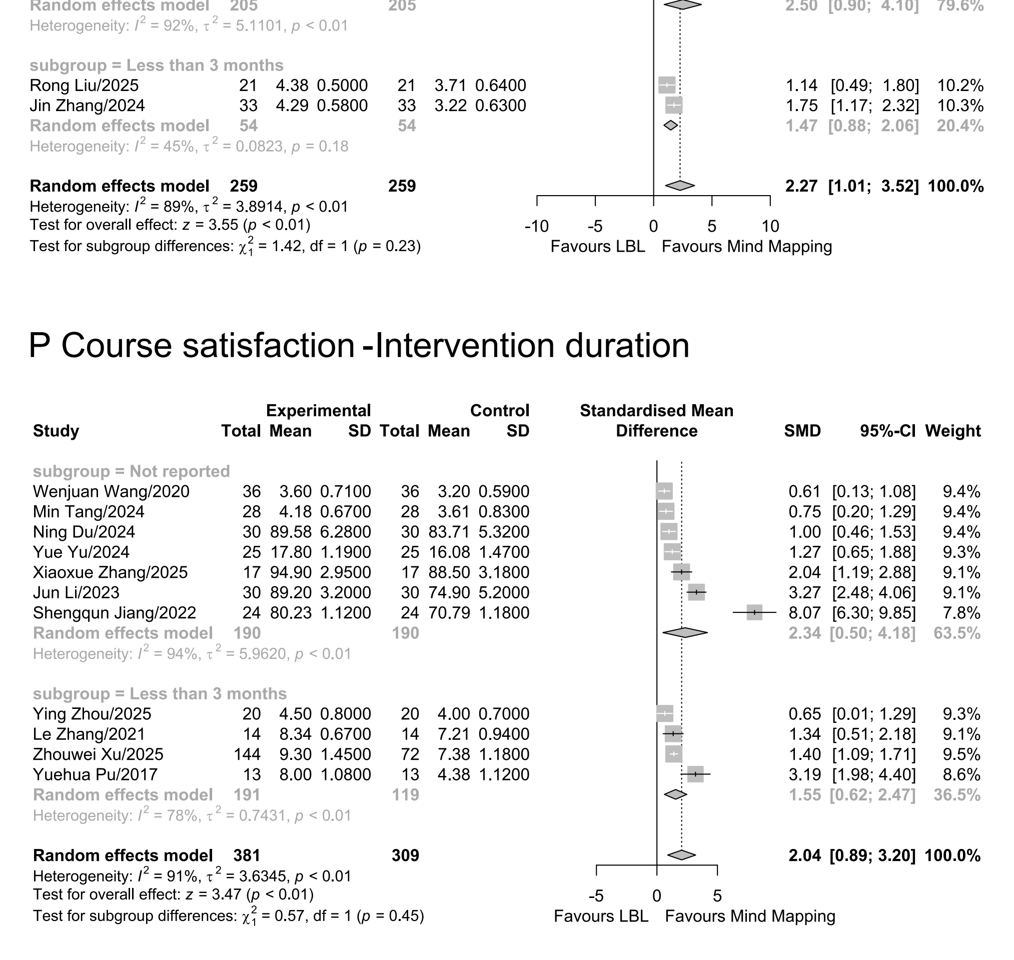

Supplement: Supplementary Figure S4 — Subgroup meta-analyses of different departments and teaching methods on questionnaire surveys results. The meta-analyses on the effect of mind mapping with the subgroup analyses of different departments on (A) clinical reasoning, (B) learning motivation, (C) problem-solving ability and (D) course satisfaction; The meta-analyses on the effect of mind mapping with the subgroup analyses of only mind mapping group and mind mapping combined with other teaching methods on (E) clinical reasoning, (F) learning motivation, (G) problem-solving ability and (H) course satisfaction; The meta-analyses on the effect of mind mapping with the subgroup analyses of different methods of generating mind map on (I) clinical reasoning, (J) learning motivation, (K) problem-solving ability and (L) course satisfaction; The meta-analyses on the effect of mind mapping with the subgroup analyses of different intervention duration on (M) clinical reasoning, (N) learning motivation, (O) problem-solving ability and (P) course satisfaction. The large diamond at the bottle of the plot represents the pooled SMD of all studies. The width of the diamond represents with 95%CI. [file Image_4.pdf]
