# Supplementary material for: The effectiveness of mind mapping versus lecture-based learning in medical education of China’s standardized residency training: a systematic review and meta-analysis of randomized controlled studies
Source: Front Med (Lausanne). 2026 May 5;13:1789650. doi: 10.3389/fmed.2026.1789650 (PMC13183817; doi:10.3389/fmed.2026.1789650)

A Level of theoretical knowledge

Study	Experimental Events	Experimental Total	Control Events	Control Total	OR	95%-CI	Weight
Xin Liao/2022	40	40	33	40	18.13	[1.00; 329.27]	2.8%
Yan Xu/2018	11	15	4	15	7.56	[1.50; 38.15]	9.0%
Lin Guo/2025	27	32	18	32	4.20	[1.29; 13.70]	16.8%
Yanming Zhang/2024	27	40	20	40	2.08	[0.84; 5.14]	28.6%
Jingmin Dong/2023	18	20	10	20	9.00	[1.64; 49.45]	8.1%
Hongyan Li/2022	14	19	9	22	4.04	[1.07; 15.27]	13.3%
Li Yao/2019	58	63	49	62	3.08	[1.03; 9.24]	19.4%
Dongzhu Zhang/2019	4	5	0	5	33.00	[1.06; 1023.56]	2.0%
**Random effects model**		**234**		**236**	**3.91**	**[2.26; 6.78]**	**100.0%**

Heterogeneity: $I^2 = 0\%$, $\tau^2 = 0$, $p = 0.52$
Test for overall effect: $t_7 = 5.87$ ($p < 0.01$)

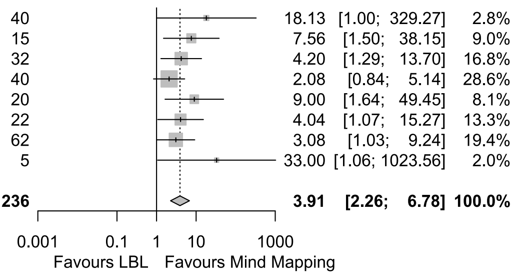

B Clinical reasoning

Study	Experimental Events	Experimental Total	Control Events	Control Total	OR	95%-CI	Weight
Shuo Wu/2022	20	23	13	23	5.13	[1.18; 22.24]	9.1%
Yan Xu/2018	12	15	5	15	8.00	[1.52; 42.04]	7.1%
Jinyu Zhang/2025	28	35	11	24	4.73	[1.49; 14.98]	14.8%
Lin Guo/2025	27	32	16	32	5.40	[1.66; 17.56]	14.1%
Jingmin Dong/2023	19	20	11	20	15.55	[1.73; 139.65]	4.1%
Hongyan Li/2022	17	19	12	22	7.08	[1.31; 38.33]	6.9%
Bing Zhou/2021	42	49	31	49	3.48	[1.30; 9.36]	20.1%
Li Yao/2019	55	63	47	62	2.19	[0.86; 5.63]	22.1%
Dongzhu Zhang/2019	4	5	0	5	33.00	[1.06; 1023.56]	1.7%
**Random effects model**		**261**		**252**	**4.46**	**[2.85; 6.97]**	**100.0%**

Heterogeneity: $I^2 = 0\%$, $\tau^2 = 0$, $p = 0.66$
Test for overall effect: $t_8 = 7.71$ ($p < 0.01$)

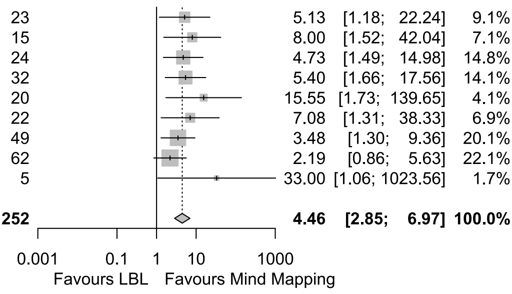

C Learning motivation

Study	Experimental Events	Experimental Total	Control Events	Control Total	OR	95%-CI	Weight
Shuo Wu/2022	21	23	14	23	6.75	[1.26; 36.03]	5.8%
Xin Liao/2022	39	40	31	40	11.32	[1.36; 94.25]	3.7%
Jinyu Zhang/2025	27	35	12	24	3.38	[1.10; 10.38]	11.8%
Lin Guo/2025	28	32	17	32	6.18	[1.76; 21.71]	9.7%
Yanming Zhang/2024	26	40	23	40	1.37	[0.56; 3.39]	16.9%
Jingmin Dong/2023	18	20	11	20	7.36	[1.34; 40.55]	5.6%
Bing Zhou/2021	43	49	31	49	4.16	[1.48; 11.69]	13.6%
Li Yao/2019	52	63	35	62	3.65	[1.60; 8.29]	19.5%
Jianping Gong/2023	21	30	10	30	4.67	[1.57; 13.87]	12.4%
Dongzhu Zhang/2019	5	5	0	5	121.00	[2.02; 7259.18]	1.0%
**Random effects model**		**337**		**325**	**3.95**	**[2.43; 6.42]**	**100.0%**

Heterogeneity: $I^2 = 14\%$, $\tau^2 = 0.0586$, $p = 0.31$
Test for overall effect: $t_9 = 6.38$ ($p < 0.01$)

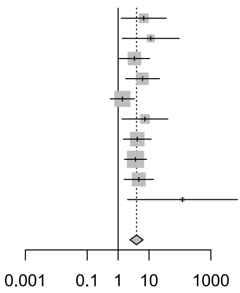

D Autonomous learning ability

Study	Experimental Events	Experimental Total	Control Events	Control Total	OR	95%-CI	Weight
Xin Liao/2022	40	40	32	40	21.18	[1.18; 380.90]	3.9%
Jinyu Zhang/2025	26	35	11	24	3.41	[1.13; 10.30]	26.4%
Hongyan Li/2022	16	19	11	22	5.33	[1.20; 23.66]	14.5%
Bing Zhou/2021	44	49	30	49	5.57	[1.88; 16.56]	27.1%
Jianping Gong/2023	23	30	11	30	5.68	[1.84; 17.49]	25.4%
Dongzhu Zhang/2019	4	5	0	5	33.00	[1.06; 1023.56]	2.7%
**Random effects model**		**178**		**170**	**5.40**	**[3.16; 9.24]**	**100.0%**

Heterogeneity: $I^2 = 0\%$, $\tau^2 = 0$, $p = 0.76$
Test for overall effect: $t_5 = 8.08$ ($p < 0.01$)

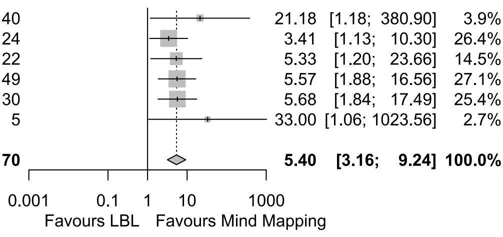

E Problem solving ability

Study	Experimental Events	Experimental Total	Control Events	Control Total	OR	95%-CI	Weight
Yanming Zhang/2024	30	40	25	40	1.80	[0.69; 4.70]	45.8%
Jianping Gong/2023	24	30	13	30	5.23	[1.66; 16.51]	38.1%
Xin Liao/2022	39	40	32	40	9.75	[1.16; 82.11]	16.1%
**Random effects model**		**110**		**110**	**3.55**	**[0.48; 26.27]**	**100.0%**

Heterogeneity: $I^2 = 36\%$, $\tau^2 = 0.2712$, $p = 0.21$
Test for overall effect: $t_2 = 2.72$ ($p = 0.11$)

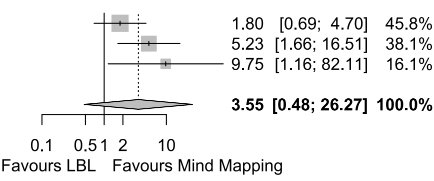

F Proficiency in literature retrieval

Study	Experimental Events	Experimental Total	Control Events	Control Total	OR	95%-CI	Weight
Yanming Zhang/2024	32	40	23	40	2.96	[1.09; 8.01]	35.6%
Li Yao/2019	57	63	39	62	5.60	[2.09; 15.02]	36.4%
Shuo Wu/2022	21	23	14	23	6.75	[1.26; 36.03]	12.6%
Jingmin Dong/2023	17	20	8	20	8.50	[1.86; 38.82]	15.3%
**Random effects model**		**146**		**145**	**4.87**	**[2.35; 10.08]**	**100.0%**

Heterogeneity: $I^2 = 0\%$, $\tau^2 = 0$, $p = 0.64$
Test for overall effect: $t_3 = 6.92$ ($p < 0.01$)

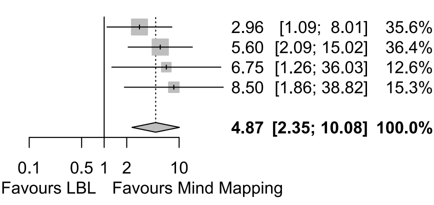

G Clinical skills

Study	Experimental Events	Experimental Total	Control Events	Control Total	OR	95%-CI	Weight
Bing Zhou/2021	41	49	30	49	3.25	[1.25; 8.40]	37.2%
Jinyu Zhang/2025	28	35	12	24	4.00	[1.26; 12.65]	25.4%
Lin Guo/2025	27	32	16	32	5.40	[1.66; 17.56]	24.2%
Xin Liao/2022	38	40	30	40	6.33	[1.29; 31.11]	13.3%
**Random effects model**		**156**		**145**	**4.23**	**[2.67; 6.71]**	**100.0%**

Heterogeneity: $I^2 = 0\%$, $\tau^2 = 0$, $p = 0.87$
Test for overall effect: $t_3 = 9.96$ ($p < 0.01$)

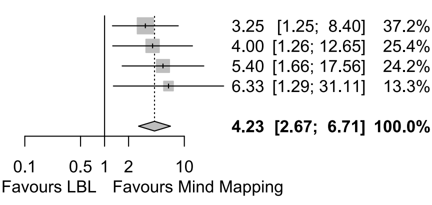

H course satisfaction

Study	Experimental Events	Experimental Total	Control Events	Control Total	OR	95%-CI	Weight
Yunfeng Jiang/2024	28	30	21	30	6.00	[1.17; 30.72]	7.1%
Shanshan Cui/2024	22	23	17	23	7.76	[0.85; 70.75]	3.9%
Hongjuan Shi/2023	26	30	19	30	3.76	[1.04; 13.65]	11.4%
Shuo Wu/2022	20	23	15	23	3.56	[0.80; 15.72]	8.6%
Yan Xu/2018	13	15	6	15	9.75	[1.59; 59.70]	5.8%
Qian Liu/2025	23	23	17	23	17.46	[0.92; 330.96]	2.2%
Lin Guo/2025	25	32	14	32	4.59	[1.54; 13.67]	15.9%
Jin Zhang/2024	31	33	25	33	4.96	[0.97; 25.48]	7.1%
Yanqian Deng/2024	49	50	43	50	7.98	[0.94; 67.46]	4.2%
Wenjie Xu/2024	28	30	21	30	6.00	[1.17; 30.72]	7.1%
Fan Liu/2023	16	16	10	16	20.43	[1.04; 401.67]	2.1%
Bing Zhou/2021	45	49	34	49	4.96	[1.51; 16.31]	13.4%
Shuai Fu/2021	15	15	13	15	5.74	[0.25; 130.37]	1.9%
Yao Hu/2025	27	30	20	30	4.50	[1.09; 18.50]	9.5%
**Random effects model**		**399**		**399**	**5.38**	**[4.30; 6.74]**	**100.0%**

Heterogeneity: $I^2 = 0\%$, $\tau^2 = 0$, $p = 1.00$
Test for overall effect: $t_{13} = 16.24$ ($p < 0.01$)

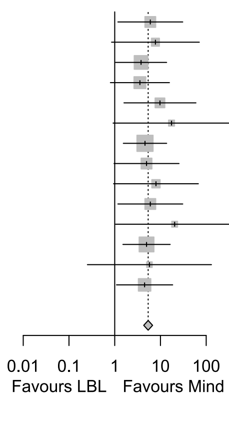

Supplement: Supplementary Figure S5 — Forest plots of binary endpoints of questionnaire surveys results. The meta-analyses on the effect of mind mapping on level of (A) theoretical knowledge, (B) clinical reasoning, (C) learning motivation, (D) autonomous learning ability, (E) problem-solving ability, (F) proficiency in literature retrieval, (G) clinical skills and (H) course satisfaction. The large diamond at the bottle of the plot represents the pooled OR of all studies. The width of the diamond represents with 95%CI. [file Image_5.pdf]
